# Multi‐Component Collaborative Step‐by‐Step Coloring Strategy to Achieve High‐Performance Light‐Responsive Color‐Switching

**DOI:** 10.1002/advs.202103309

**Published:** 2021-11-21

**Authors:** Zhen Du, Ting Zhang, Hanqi Gai, Lan Sheng, Yu Guan, Xiaojun Wang, Tianyou Qin, Minjie Li, Shuo Wang, Yu‐Mo Zhang, Hui Nie, Sean Xiao‐An Zhang

**Affiliations:** ^1^ State Key Lab of Supramolecular Structure and Materials College of Chemistry Jilin University Changchun 130012 China; ^2^ School of Materials Science and Engineering Dongguan University of Technology Guangdong 523710 China; ^3^ College of Basic Medicine Jilin University Changchun 130012 China; ^4^ College of Chemistry Huazhong University of Science and Technology Wuhan 430074 China

**Keywords:** controllable coloration, ideal comprehensive performance, light‐responsive color‐switching, photoinduced proton transfer, visible‐light‐responsive color‐switching

## Abstract

Light‐responsive color‐switching materials (**LCM**s) are long‐lasting hot fields. However, non‐ideal comprehensive performance (such as color contrast and retention time cannot be combined, unsatisfactory repeatability, and non‐automated coloring mode) significantly hinder their development toward high‐end products. Herein, the development of **LCM**s that exhibit long retention time, good color contrast, repeatability, and the property of automatic coloring is reported. The realization of this goal stems from the adoption of a bio‐inspired multi‐component collaborative step‐by‐step coloring strategy. Under this strategy, a conventional one‐step photochromic process is divided into a “light+heat” controlled multi‐step process for the fabrication of the desired **LCM**s. The obtained **LCM**s can effectively resist the long‐troubled ambient‐light interference and avoid its inherent yellow background, thereby achieving the longest retention time and good repeatability. Multiple colors are generated and ultra‐fast imaging compatible with the laser‐printing technology is also realized. The application potential of the materials in short‐term reusable identity cards, absorptive readers, billboards, and shelf labels is demonstrated. The results reported herein can potentially help in developing and designing various high‐performance, switchable materials that can be used for the production of high‐end products.

## Graphical abstract

High‐performance light‐driven color‐switching material (**LCM**) is developed exploiting the step‐controllable light‐induced proton transfer process occurring between highly sensitive photoacids and “inert” acidochromic dyes. The excellent performance of the fabricated **LCM** makes it a suitable candidate that can be used for the development of reusable display and erasable ink.

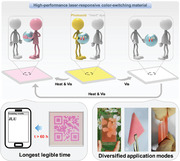

## 1 Introduction

Stimulus‐responsive color‐switching materials have attracted immense attention as they can be used to fabricate novel sensors,^[^ ^1^, ^2^, ^3^, ^4^ ^]^ display units,^[^ ^5^, ^6^ ^]^ and products of daily use.^[^ ^7^, ^8^, ^9^ ^]^ Light‐responsive color‐switching materials (**LCM**s) are currently the most popular among all the stimuli‐responsive (such as heat‐,^[^ ^10^, ^11^, ^12^ ^]^ light‐,^[^ ^13^, ^14^, ^15^ ^]^ water‐,^[^ ^16^, ^17^ ^]^ electricity‐,^[^ ^18^, ^19^ ^]^ force‐,^[^ ^20^, ^21^, ^22^ ^]^ magnetism^[^ ^23^ ^]^‐responsive) materials. This is mainly because light is a form of clean and non‐contact stimulus, and the wavelength and intensity of light can be readily tuned in time and space.

Over the past few decades, various **LCM**s have been developed such as organic **LCM**s (such as photochromic molecular switches^[^ ^24^, ^25^, ^26^, ^27^, ^28^, ^29^, ^30^, ^31^, ^32^, ^33^ ^]^ and photoinduced proton transfer systems^[^ ^34^, ^35^, ^36^, ^37^, ^38^ ^]^), inorganic nanocomposites,^[^ ^39^, ^40^ ^]^ and organic–inorganic composites (such as TiO_2‐x_‐based nanocrystals/redox dyes, SnO_2‐x_‐based nanocrystals/redox dyes,^[^ ^41^, ^42^, ^43^, ^44^, ^45^ ^]^ Au nanocrystals/molecular switches,^[^ ^46^, ^47^ ^]^ polyvinylpyrrolidone (PVP) or polyethylene terephthalate (PET)/WO_3_ hybrid membranes,^[^ ^48^, ^49^ ^]^ gelatin & polyvinyl alcohol —or PVP/polyoxometalate‐based hybrids,^[^ ^50^, ^51^ ^]^ and metalorganic frameworks^[^ ^52^ ^]^). These pioneering efforts have resulted in the significant advancement of the field. However, there are some troublesome issues that need to be addressed. The materials exhibit short color retention time (or optical memory) (the retention time ranges between a few minutes to a few hours) and slow response and/or poor reversibility in solid media. The level of color contrast achieved is unsatisfactory, and the generation of a background of undesirable color is observed. Automatic and digital imaging/coloring cannot be achieved using these materials. These factors hinder the development of high‐end products (such as rewritable displays, medical sensors, industrial sensors, information storage devices,^[^ ^53^, ^54^ ^]^ energy storage materials,^[^ ^55^, ^56^ ^]^ and molecular electronic devices^[^ ^57^, ^58^ ^]^).

When the mechanism of coloration was studied for various photoreversible materials, we observed that in most cases, the mechanism involved a single‐step (e.g., in photochromic switches) or an approximate “one‐step” stimulus‐response mode. The latter can be observed during the process of photoinduced proton transfer and in photocatalytic redox systems. The conventional “one‐step” stimulus‐responsive process is a simple mode that results in the generation of rapid responses. However, uncontrolled and unexpected color changes could be observed under this mode, resulting in the non‐ideal comprehensive‐performance of the materials. Various switchable materials that functioning though the “one‐step” stimulus‐response mode suffer from uncontrolled color changes. Therefore, it is important to develop methods that can be followed for the fabrication of stimulus‐responsive materials to gain complete human control.

Various chemical processes that are difficult to realize following traditional artificial methods are observed in biochemical systems. The complex one‐step processes followed by human beings to conduct chemical processes are split by biological systems into multi‐step processes that can be tuned and controlled. Each of these steps can be executed in an orderly and accurate manner. The efficiency of the process is closely related to collaborative interactions between the constituent units (containing reaction units/subunits (molecules/atomic‐groups/ions) and surrounding microenvironment). Mutualism helps avoid the formation of defects and achieve goals that cannot be otherwise achieved by the individual constituents. The law of natural development reveals that the benefits achieved by a multi‐member system are significantly greater than the benefits achieved by a single system fighting alone.^[^ ^59^ ^]^ We analyzed the mode of functioning of natural systems and hypothesized that the multi‐component collaborative controllable “dual/multi‐step” stimulus‐response mode (observed in multi‐component supramolecular systems) could potentially help in fabricating ideal high‐performance color‐switching materials.

Herein, we attempted to split the conventional light‐driven “one‐step” coloring process into a multi‐step controllable process driven by light and heat by adjusting the extent of synergism between the different components. The inter‐/intra‐molecular kinetics and thermodynamics of the controllable steps have been presented. The process is used to successfully impart the **LCM**s with the properties of long retention time (>60 h), high color contrast and good repeatability (>20 cycles) Multiple colors with ideal white as the background, and ultra‐fast automatic imaging compatible with digital laser printing technology are also realized. These properties make the fabricated color‐switching systems promising candidates that can be used for the fabrication of short‐term reusable display systems. The results obtained following the strategy of multi‐component collaborative controllable “dual/multi‐step” stimulus‐responsive mode provide new ideas or perspectives that can help address the problems faced during the fabrication and use of other stimulus‐response systems and/or materials.

## 2 Results and Discussion

### 2.1 Molecular Design and Mechanism Followed to Achieve Controllable Multi‐Step Coloration

The process of photoinduced proton transfer (PPT) is widely observed in living systems (e.g., in bacteriorhodopsin^[^ ^60^ ^]^). The process has been widely studied over the years.^[^ ^61^, ^62^ ^]^ The process of artificial PPT (observed in the presence of metastable photoacids/dyes) has been recently used for the fabrication of **LCM**s.^[^ ^35^, ^36^ ^]^ The input visible light is used to convert the traditional acid‐triggered chemical processes to easy‐to‐control photochemical reaction processes without the need to introduce foreign chemicals. The process is characterized by good reversibility.^[^ ^34^ ^]^ Because the amount of energy carried by visible light is lower than the amount of energy carried by ultraviolet light (UV), under these conditions, less damage is caused to organic molecules. A multiple colors expansion can be achieved as a wide range of dyes can be used to fabricate the materials. At the same time, a high contrast ratio for coloration and a positive imaging mode can be realized. Theoretically, the PPT process primarily consists of two steps: 1) the step involving the release of protons from photoacids under conditions of light irradiation and 2) the step involving the acid‐induced coloration of dyes. The two processes occur rapidly and are similar in terms of reaction rates. Hence, the process functions as a “one‐step” photochromic process.^[^ ^34^, ^35^, ^36^ ^]^ The **LCM** fabricated using metastable photoacids/dyes is characterized by an intrinsic yellow background (generated by the photoacid) and a short optical memory (<20 min without protection layer, or ≈5 h in the presence of a light‐shielding layer^[^ ^34^ ^]^) (Figure, bottom row).

**Figure 1:**
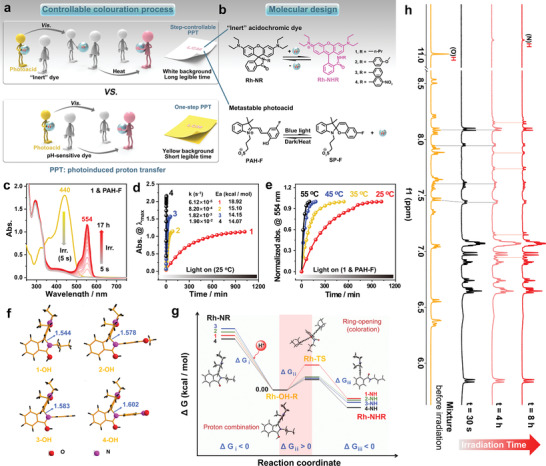
Schematic illustrations, molecular design, and proof of mechanism for the process of controllable coloration in solution. a) Schematic illustrations of different mechanisms of action of photoinduced proton transfer (PPT)‐based **LCM**s (previously reported and reported herein). b) Molecular structures and the corresponding isomerization processes of the “inert” acidochromic dyes (Rh‐NR) and metastable photoacid (PAH‐F). c) UV–vis spectral profiles recorded for **1** and PAH‐F (10 equivalents) in methanol (MeOH) at ambient temperature under conditions of varying irradiation times (0 s, 5 s, and 17 h). d) Changes in the absorbance at *λ*_max_ recorded for **1–4** (0.025 × 10^−3^ m, MeOH) in the presence of PAH‐F (10 equivalents; under conditions of blue light irradiation) recorded as a function of time at 25 ℃. Inset shows the rate constants and the corresponding apparent activation energies. e) Changes in the normalized UV–vis absorbance spectral profiles recorded for **1** at 554 nm (0.025 × 10^−3^ m, MeOH) in the presence of PAH‐F (10 equivalents; under conditions of blue light irradiation) recorded as a function of time at different temperatures. f) Calculated structures of the four intermediates (Rh‐OH‐Rs) and the corresponding C(sp^3^)—N bond lengths marked nearby. g) Optimized structures obtained during the protonation of **1**–**4** and the corresponding free energies (structural changes in **1** is a representative example). h) ^1^H NMR spectral profiles (500 MHz, DMSO‐d_6_) recorded for **4** and PAH‐F (2 equivalents), recorded under conditions of varying irradiation times (0 s, 30 s, 4 h, and 8 h).

We attempted to decelerate the rate of protonation of the dye to such an extent that the rate of protonation of the dye was significantly slower than the rate of release of proton observed in the photoacid using highly “sensitive” photoacids (PAH‐F) (Figure S1 and Table S1, Supporting Information) and “inert” acidochromic dyes (Rh‐NRs; **1**–**4**) to address these issues. The structures and processes of isomerization of the Rh‐NRs (upon addition of acid) and PAH‐F (under conditions of irradiation with visible light) are presented in Figure 1b. The structurally variable polyethylene glycol (PEG) unit, a phase‐change material containing numerous electron‐rich oxygen atoms in its flexible molecular chains, was selected for generating an “accommodative microenvironment” during the process of fabrication of the desired **LCM**. The photoacid could release protons that could be captured by the PEG units present in the vicinity under conditions of visible light irradiation. The PEG‐covered “inert dye” maintains a colorless state, enabling the generation of a white background. The “inert dye” will be colored only when visible light is irradiated and additional energy is introduced into the system in the form of heat. Self‐coloration of the background can be avoided under conditions of ambient light and a prolonged time of coloration can be achieved (Figure 1a, upper row).

Photophysical/chemical processes involving compounds **1–4** and PAH‐F were investigated in the solution state. The process involving a mixture of **1** and PAH‐F was used as a representative example (Figure 1c). When **1** and PAH‐F were dissolved in methanol (0.025 × 10^−3^ _M_), a yellow solution was formed. The absorption spectral profile for the solution was recorded, and it was observed that the absorption peak appeared at ≈440 nm. The position of the absorption peak was comparable to the position of the absorption peak recorded for a solution containing PAH‐F (Figure S2a, Supporting Information). This suggested that PAH‐F primarily existed in the *trans‐*form and **1** was present in the ring‐closed form (**1C**). The solution turned colorless when irradiated with blue light (irradiation time: 5 s). Absorbance in the visible region was not observed, indicating that ring‐closing photoisomerization of PAH‐F (SP‐F) occurred in the presence of unprotonated **1**. When the illumination time was increased, the absorption peak (554 nm) corresponding to protonated **1** appeared gradually (Figure S2b, Supporting Information). It was observed that all of **1** was protonated when the time of illumination was >11 h. Similar results can be seen in the time‐varying fluorescence spectra (Figure S3, Supporting Information). This confirms that the PPT process consists of two controllable steps: 1) the rapid photoinduced proton release step involving PAH‐F and 2) the slow protonation/tautomerization step involving **1**.

Similar results were also observed when other mixtures were studied (Figure 1d). The absorbance was recorded at the maximum wavelengths for the tautomers of the protonated Rh‐NRs (i.e., Rh‐NHRs). The time required to reach equilibrium followed the order **1** > **2** > **3** > **4**. Analysis of the data collected at an early stage revealed that the process followed first‐order kinetics (Figure S4 and Note S1, Supporting Information). The apparent rate constants (*k*(s)) for the processes were also calculated (Figure 1d inset). The rates of ring‐opening for the four derivatives increased as the electron‐withdrawing power of the substituents increased. The slowest kinetics was recorded for **1** (R = *n*‐Pr). The results agreed well with the results reported by Professor Harbron and co‐workers.^[^ ^63^ ^]^ It is worth mentioning that the rates of acid‐induced ring‐opening for Rh‐NRs could be significantly accelerated by increasing the reaction temperature (Figure 1e, Figures S5–S7 and Table S2, Supporting Information). The apparent activation energies (*E*a(s)) of the protonation reactions involving **1**–**4** were determined (Figure 1d inset) using the Arrhenius formula (Figure S7 and Note S2, Supporting Information). It was observed that the *Ea* recorded for the reaction involving **1** was higher than the *Eas* recorded for the reactions involving other compounds. Hence, the rate of protonation of **1** was the slowest.

The differences in the rates of the reactions were further studied by carrying out theoretical calculations. The order of the C(sp^3^)—N bond lengths of the four intermediates (protonated closed forms of Rh‐NR, i.e., Rh‐OH‐R) was **1** (1.544 Å) < **2** (1.578 Å) < **3** (1.583 Å) < **4** (1.602 Å) (Figure 1f). This indicated that the most stable intermediate was formed for **1**. Thus, the ring‐opening for a lactam is difficult. It was also observed that the calculated combination of protons to form Rh‐OH‐R was spontaneous and the changes in the free energy (ΔG_i_) values were found to be negative. The ring‐opening step was found to be the rate‐controlling step characterized by high free energy barriers (ΔG_ii_). The order agreed well with the order determined by carrying out experiments (Figure 1g, Figure S8 and Table S3, Supporting Information).

The reaction mechanism of the steps‐controllable proton transfer process was further studied using the proton nuclear magnetic resonance (^1^H‐NMR) spectroscopy technique (Figure 1h and Figure S9, Supporting Information). Considering that the protonation rates for **1**–**3** were significantly slow, **4** was selected to conduct the ^1^H‐NMR experiments. The original mixture owned the spectrum as a mixture of PAH‐F and **4C** (Figure 1h, Mixture). As the irradiation time was increased, the signals corresponding to PAH‐F disappeared and a new set of peaks corresponding to SP‐F (30 s) appeared (Figure S9a, Supporting Information) in the spectral profiles. After 4 h of irradiation, the signals corresponding to the protonated ring‐open form of **4** (i.e., **4O**) (Figure S9b, Supporting Information) could be observed. Analysis of the signals revealed that 25.8% conversion (evaluated by integral areas) was achieved under these conditions. The extent of conversion increased to 34.3% following 8 h of irradiation. Notably, the conversion of **4O** to **4C** is reversible and the reaction can proceed backward in the dark and under conditions of heat (Figure S10, Supporting Information). These results indicated that the reverse proton transfer reaction could occur in the dark or when heat was applied (Figure S11 and Note S3, Supporting Information). This can be attributed to the fact that PAH‐F is the most favorable thermodynamic structure of the photoacid in the dark and heat accelerates the process of proton recapture, resulting in the reverse transformation of SP‐F (to PAH‐F) and **4O** (to **4C**) (Figure S12, Supporting Information).

### 2.2 Steps‐Controllable Coloration of LCM on Solid Substrates

**Figure 2:**
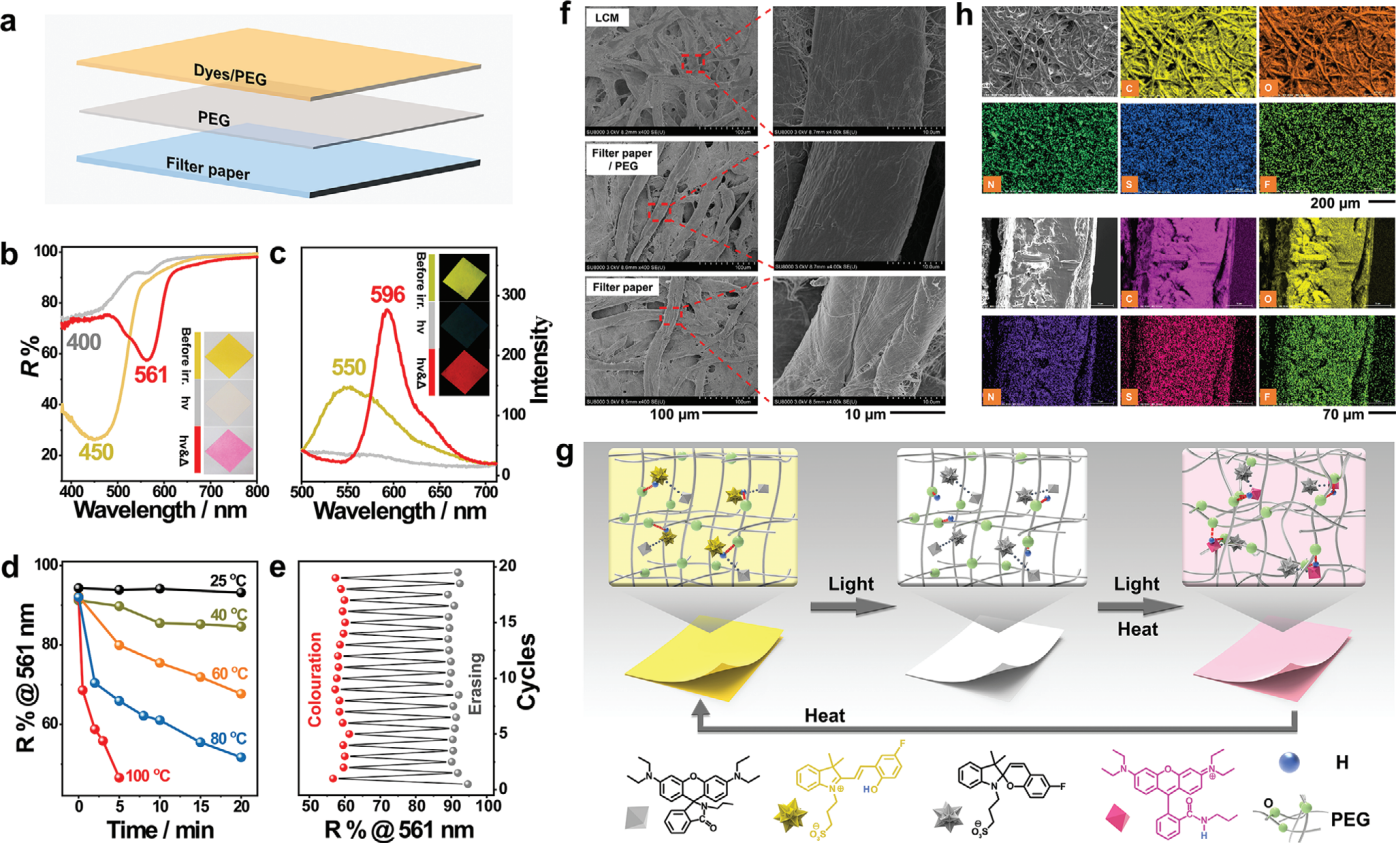
Performances and schematic representation of the mechanism of action of the controllable **LCM**s in solid matrices. a) Schematic representation of the three‐layered structure of **LCM**s. b) Reflective UV–vis spectral profiles and c) fluorescent spectral profiles recorded for the **LCMs** before irradiation, after irradiation, and under conditions of simultaneous irradiating and heating. The images in the inset present the corresponding changes in color. d) Changes in the reflectivity at 561 nm recorded for the **LCM** as a function of time under conditions of continuous irradiation at varying heating temperatures. e) Reflectance recorded during the 20 consecutive coloring (red sphere)–erasing (gray sphere) cycles (yellow–white–pink) at 561 nm. f) Images recorded using the scanning electron microscopy (SEM) technique for the interfaces of **LCM**, filter paper/PEG, and filter paper and the corresponding magnified SEM images. g) Molecular schematic of the controllable coloration processes of **LCM**. h) SEM and the corresponding EDS mapping images recorded for surfaces and cross‐sections of the **LCM**.

**LCM** fabricated with a three‐layered structure consisting of paper substrate, PEG and PEG containing scattered PAH‐F & **1** was used for the experiments (Figure). The changes in the color and the spectral profiles were similar to the changes in the color and spectral profiles recorded for the sample in the solution state (Figure 2b,c). Before irradiation, the color/emission recorded for the as‐prepared paper was yellow/yellow‐green. The reflective/fluorescent broadband was centered at 450/550 nm (**yellow lines**). The positions were similar to the positions recorded for the overlay of PAH‐F and **1**. Following the irradiation with blue light (irradiation time: 30 s), the color/emission appeared white/dark. Reflective/emission bands were not observed above 500 nm (**gray lines**), indicating the ring‐closing photoisomerization of PAH‐F (i.e., SP‐F) while **1** kept in **1C**. When the yellow paper or the irradiated white paper was irradiated with blue light under heating conditions (at 80 ℃), the generation of pink/orange‐red hue was observed. The generation of a new reflective/emission peak at 561/596 nm (**red lines**), ascribed to **1O**, was also observed. The speed of coloration increased with an increase in the heating temperature (Figure 2d). These data demonstrated that the steps‐controllable coloration involving PAH‐F and **1** can occur on solid substrates. When the substrates were heated (e.g., heated at 70 ℃ for 3 min) or kept in the dark for a period of time, the pink paper exhibiting orange‐red emission reverted to its initial yellow state. The color emitted by the sample under these conditions was the same as the color emitted by the untreated sample. The reversibility in the properties of the fabricated **LCM** was further investigated, and it was observed that the samples exhibited good reversibility even at the end of 20 successive coloring–erasing cycles (Figure 2e). The reversibility is highly correlated with the amount of PEG and the way it is added (Figure S13, Supporting Information). The scanning electron microscopy (SEM) technique was used to analyze the samples. Analysis of the images revealed that the microfibers corresponding to **LCM** were thicker (110 µm vs 89 µm), smoother, and denser than the microfibers of the untreated paper fibers (Figure 2f, and Table S4, Supporting Information). This illustrated that the introduction of PEG resulted in a significant change in the surface structure and properties of the microfibers of the paper. Its quantity is dominant, compared with dyes and PAH‐F. It was observed that PEG could effectively block the negative effects generated by the polyhydroxyl units (Figure S14, Supporting Information) and provide an “accommodative microenvironment” to the dyes and PAH‐F units to achieve controllable coloration.

The mechanism of controllable coloration for the solid **LCMs** fabricated using PAH‐F, **1**, and PEG was determined by analyzing the experimentally obtained results. A schematic representation of the mechanism has been presented in Figure 2g. At the start of the experiment, yellow PAH‐F and colorless **1C** are evenly and closely distributed on PEG. This was validated by the fact that the elements N and O (representing dye **1**) and S and F (representing PAH‐F) were found to be uniformly distributed (Figure 2h). The uniform distribution can be attributed to their structural similarity. Thus, the **LCM**s initially appear yellow (left). Following the irradiation with blue light, colorless SP‐F was formed from PAH‐F. The process was accompanied by the release of a proton, which was accepted by and temporarily stored in the surrounding PEG units. The processes of proton transfer and protonation and the change in color of the **1** units present in the vicinity were significantly hindered by the PEG barrier and the high activation energy of the ring‐opening reaction. This ensured the generation of white **LCM**s (middle). When the **LCM** samples were simultaneously heated and illuminated with blue light, the thermo‐energy increased. This significantly promoted the processes of activation and dynamic adjustment corresponding to the phase change of the PEG and the structures of **1** and SP‐F. Under these conditions, intra‐/inter‐molecular associations weakened. These resulted in the acceleration of the charge transfer of the proton and counter ion (i.e., –SO_3_^–^) in SP‐F to highly close to **1**. The tautomerization of **1** and stabilization of **1O** were promoted in the presence of PEG units. The generation of multiple supramolecular interactions between the polyether subunits and **1O** helped in the process. As a result, the **LCM**‐based paper appeared pink (right). When the **LCM** was heated and then cooled, PAH‐F in it rapidly transformed to its ring‐open form by accepting a proton. The process was assisted by the thermo‐activated structural alteration of PEG. Compound **1** was dynamically separated from PAH‐F, resulting in the instant formation of the colorless **1C** from the temporary tautomeric structure of **1O**. Thus, controllable coloration and reversible erasure of the properties of **LCM** were realized. Self‐coloration of the background under ambient light conditions can be avoided as the imaging process proceeds under high‐energy (input) conditions. Subsequently, the time of coloration could be significantly increased and the issues pertaining to the yellow background of **LCM** prints were addressed. PEG units functioned as substrate passivators, temporary proton‐storage media, proton transfer units, and stabilizers as they played an “accommodative” role. The results were also validated by the fact that solutions of PAH‐F and **1** in dimethylsulfoxide (DMSO), a good hydrogen bond acceptor but a bad stabilizer that exhibited high degrees of flexibility, did not turn pink when continuously exposed to blue light. The solution did not turn pink even when chemical acids were introduced into the systems (Figure S15, Supporting Information).

**Figure 3:**
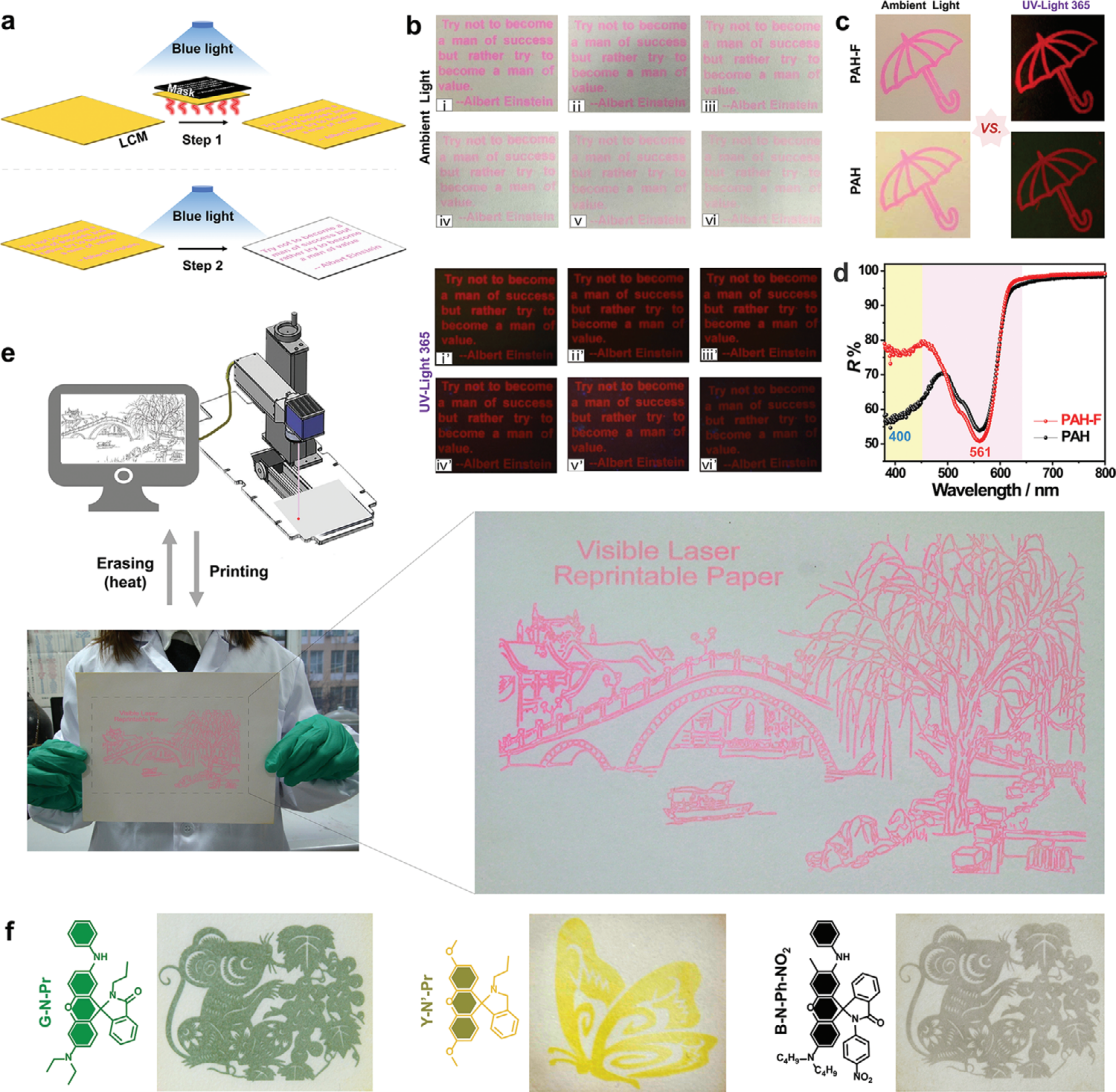
Patterning mode, photochromic performance, and multicolor property of **LCM**. a) Schematic illustrations of the photomask printing processes involving **LCM**. b) Visible and fluorescent images recorded for **LCM** under conditions of ambient air following the process of writing (time: 0.5, 12, 24, 36, 48, and 60 h). c) Visible and fluorescent images printed on **LCM** based on PAH & **1** and PAH‐F & **1**. d) Corresponding reflective spectral profiles of pink images observed on the two **LCM**s. e) Schematic illustration of the digital laser printing process involving **LCM** and the image of an A4 size print. f) Multicolor ink‐free laser prints and corresponding structures of “inert” acidochromic dyes used.

The as‐prepared **LCM**s, in the presence of a photomask, can be used to generate information under conditions of “blue light + heat”. The simultaneous blue light‐ and heat‐treatment condition is followed by conditions of blue light scanning (Figure). Pink letters on white paper were observed and fluorescent images were recorded. A good contrast ratio was obtained after 60 h (≈2 days) of treatment under ambient light conditions (Figure 3b). The retention time was further determined according to the intelligent recognition process of a printed QR code pattern (Figure S16, Supporting Information). It was observed that the newly synthesized PAH‐F, the photoinduced proton release capability of which was higher than that of the photoacid without fluorine substituent (PAH), could be effectively used to achieve better color contrast in images (Figure 3c). The high photoinduced proton release ability could be attributed to the fact that a low concentration of the substance affected the process of *trans–cis* isomerization (Figure S17, Supporting Information). Analysis of the reflective spectral profiles recorded for the samples revealed that the former exhibited a higher reflectance at ≈400 nm and a lower reflectance at ≈561 nm than the latter (Figure 3d).

Fortunately, these complex processes could be simplified and significantly accelerated following the processes of laser printing, which integrates the properties of light and heat (Figure S18, Supporting Information). As shown in Figure 3e, an automatic laser printer can be used to rapidly print A4‐sized, complex, and fine patterns. Tiny squares (1.5 × 1.5 mm^2^) inside printed letters (Figure S19, Supporting Information) and numbers characterized by font sizes in the range of 4–22 pts could be clearly observed (Figure S20, Supporting Information). These results reveal that **LCM** is fully compatible with laser printing technology, and legible images with excellent resolution can be produced. Similar results were also obtained using the laser printing techniques when green, yellow, and black colors were printed on **LCM**‐based papers using “inert” acidochromic dyes (Figure 3f and Figure S21, Supporting Information). This kind of multi‐color display is the most likely solution to achieve full‐color system in addition to structural colors.^[^ ^64^, ^65^, ^66^, ^67^, ^68^ ^]^

### 2.3 Potential Applications of LCMs

The mode of application of the material dictates its applicability. The material can be used to conduct the dip‐coating method. The PAH‐F & **1** system present in the polymer solutions of PEO/PEG can be used as “paint” or “ink.” Various matrix‐based **LCM**s can be fabricated following the processes of printing/scrape coating. As shown in Figure, we could fabricate rigid glass‐based “transparent” **LCM**s and filter paper‐ and PET‐based flexible **LCM**s.

**Figure 4:**
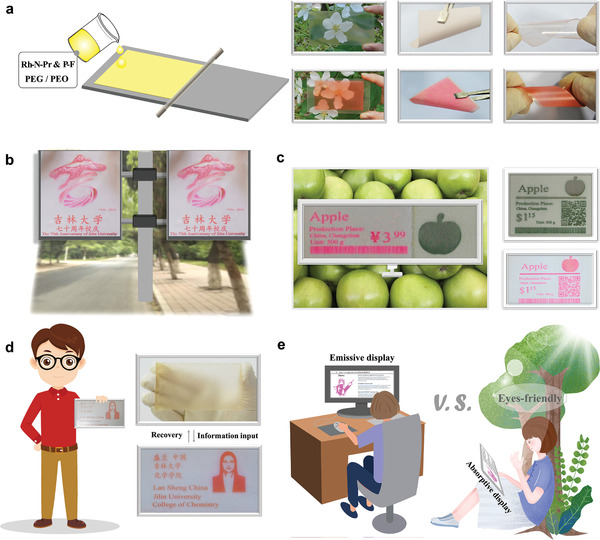
Potential applications of **LCM**s. a) Schematic illustration of the process of coating **LCM**s fabricated on different matrixes, and the photographs of **LCM**s on glass, filter paper, and PET matrices recorded before and after coloration. b) Prototype of the PET‐based **LCM** that can be used for developing billboards. c) Prototype of the **LCM** that can be used to fabricate multi‐color shelf labels used in supermarkets. d) Conceptual image representing an **LCM** coated on glass for the fabrication of short‐term ID cards and the automatic laser printing and erasing of ID‐card images. e) Application prospect of **LCM**s for the fabrication of printable/erasable light‐absorbing readers.

The excellent optical response characteristics exhibited by **LCM**s, the diverse application modes, and the compatibility of the **LCM**s with the laser printing technology make **LCM**s suitable candidates in the production of high‐end products such as short‐term reusable‐displays. The use of the PET‐based **LCM** exhibits for the development of short‐term reusable “billboards” can potentially help eliminate white pollution caused by a large number of single‐use billboards (Figure 4b). **LCM**s can also be used for the production of short‐term reusable‐display products, such as colorful short‐term shelf labels (Figure 4c). Rigid **LCM**s can be used for fabricating temporary passes, identity (ID) cards, and delivery notes and transport documents (Figure 4d). The used information can be “destroyed” without trace following the heat‐erasing cycle and the material can be reused. Thus, the data can also be protected from being leaked to unauthorized personnel. The use of a combination of flexible **LCM**s and laser printing technology can help convert the eye‐damaging “light‐emitting” electronic displays to “meta‐stable” eye‐friendly “light‐absorbing” readers. The technique can be used to reduce the amount of energy consumed during online reading while maintaining a good reading experience under conditions of a bright outdoor environment (Figure 4e, Figure S22, Supporting Information).

## 3 Conclusion

Using a biomimic multi‐component collaborative step‐by‐step coloring strategy, we have successfully developed a new **LCM** and product prototype that is characterized by excellent performance. The system exhibits the maximum optical memory reported to date (>60 h) (Table S5, Supporting Information), has a white background, achieves good repeatability, high resolution/contrast and multicolor (or fluorescent emission) printing. The conventional “one‐step” photoinduced proton transfer chromic processes were divided into “light+heat” controlled multi‐step processes through reasonable design of each component, especially the “inert” dye. Because the “light + heat” synergistic imaging is compatible with digital laser printing technology, the imaging speed is instantaneous. The intra‐/inter‐molecular photochemical and physical reactions and kinetic and thermodynamic control processes were studied by conducting kinetic experiments and using UV–vis, fluorescent spectroscopy, and NMR spectroscopy techniques. The samples were also analyzed using the SEM technique. Theoretical calculations were also conducted to analyze the samples. The PEG units provided an “accommodative microenvironment” and helped promote the activation, structural change, charge transfer, and stabilization processes involving the photoacids and dyes present in the **LCM**s. The results reported herein can potentially help in understanding the working mechanism of **LCM**s and promote the application of **LCM** in short‐term rewritable displays (such as confidential meeting documents, reprintable ID cards, billboards, shelf labels, and absorptive readers) and erasable ink. Furthermore, the success of this biomimetic strategy reveals that the multi‐component collaborative step‐by‐step reaction strategy can be used for designing various high‐performance switchable materials and high‐end products. This can potentially help in the advancement of the field.

## Conflict of Interest

The authors declare no conflict of interest.

## Supporting information

Supporting Information

## Data Availability

Research data are not shared.
