# Supplementary material for: Multi‐Component Collaborative Step‐by‐Step Coloring Strategy to Achieve High‐Performance Light‐Responsive Color‐Switching
Source: Adv Sci (Weinh). 2021 Nov 21;9(2):2103309. doi: 10.1002/advs.202103309 (PMC8805571; doi:10.1002/advs.202103309)
Supplement: Supplementary file 1 — Supporting Information [file ADVS-9-2103309-s001.pdf]

WILEY-VCH

ADVANCED SCIENCE
Open Access

Supporting Information

for *Adv. Sci.*, DOI: 10.1002/advs.202103309

Multi-component collaborative step-by-step coloring strategy to achieve high-performance light-responsive color-switching

Zhen Du, Ting Zhang, Hanqi Gai, Lan Sheng, Yu Guan, Xiaojun Wang, Tianyou Qin, Minjie Li, Shuo Wang, Yu-Mo Zhang, Hui Nie, and Sean Xiao-An Zhang**

Supplementary Information

Multi-component collaborative step-by-step coloring strategy to achieve high-performance light-responsive color-switching

Zhen Du[1#], Ting Zhang[2#], Hanqi Gai[1], Lan Sheng[1]*, Yu Guan[1], Xiaojun Wang[1], Tianyou Qin[3], Minjie Li[1], Shuo Wang[1], Yu-Mo Zhang[1], Hui Nie[4], and Sean Xiao-An Zhang[1]*

[1] State Key Lab of Supramolecular Structure and Materials, College of Chemistry, Jilin University, Changchun, 130012 (People's Republic of China)

[2] School of Materials Science and Engineering, Dongguan University of Technology, Guangdong, 523710, China

[3] College of Basic Medicine, Jilin University, Changchun, 130012, China

[4] Department of Chemistry and Biochemistry, University of California-Santa Barbara, Santa Barbara, California, 93106, United States

[#] Authors contributed equally to this work

Correspondence and requests for materials should be addressed to S.X.-A.Z. (seanzhang@jlu.edu.cn) or to L.S. (shenglan17@jlu.edu.cn)

Table of content

Supplementary Experimental Information 4
Supplementary Synthesis Methods 6
Supplementary Figures 12
Supplementary Notes 34
Note S1 Protonation kinetics of **1**—**4**. 34
Note S2 Ea(s) of **1**—**4**. 36
Note S3 Reversibility of **1** & PAH-F under dark or sole heating condition. 37
Supplementary Tables 38
Calculated Structures by DFT 42
Supplementary References 50

Supplementary Experimental Information

Materials. Phenylhydrazine (98%), 3-methyl-2-butanone (98%), 1,3-propanesultone (98%), niobium chloride ($NbCl_5$, 99.95%), lithium aluminium hydride ($LiAlH_4$, 97%), 3-methoxyphenol (98%), 4-fluorophenol (99%), phosphorus oxychloride ($POCl_3$, 99.5%), aniline (99.5%), n-propylamine ($NH_2CH_2CH_2CH_3$, 98%), *p*-nitroaniline (99%), methanesulfonic acid (CH_3SO_3H, 99%), acetic acid (CH_3COOH, 99.5%) and trifluoroacetic acid (TFA, 99%) were purchased from Energy Chemical (Shanghai, China). Rhodamine B, *p*-anisidine (97%) and phthalic anhydride (99%) were purchased from Aladdin (Shanghai, China). Hexamethylenetetramine (urotropine) was purchased from Beijing Chemical (Beijing, China). Acidochromic dyes: 2-Anilino-6-dibutylamino-3-methylfluoran (ODB-2) was purchased from Deba Chemical (Shanghai, China) and hot sensitive green (TFG) was purchased from Alligator Reagent (Nanjing, China). Soldium hydroxide (NaOH), sodium carbonate (Na_2CO_3), anhydrous sodium sulfate (Na_2SO_4), sulfuric acid (H_2SO_4, 95%-98%) and hydrochloric acid (HCl) were purchased from Beijing Chemical Company. 1,2-dichloroethane ($ClCH_2CH_2Cl$) was purchased from Guangfu Fine Chemical Research Institute (Tianjin, China). Triethylamine (Et_3N) was purchased from Xilong Chemical (Guangdong, China). Solvents: methanol (MeOH, HPLC) and acetonitrile (CH_3CN, HPLC) were purchased from Yuwang Group (Shandong, China). Ethanol (EtOH), *n*-hexane (Hex), ethyl acetate (EtOAc), dichromethane (CH_2Cl_2), dimethyl sulfoxide (DMSO), tetrahydrofuran (THF) and diethyl ether (Et_2O) were purchased from Beijing Chemical. Water (H_2O) here used was Wahaha purified H_2O (Hangzhou, China). PEG 20000 (molecular weight: 17,000–22,000) was purchased from Guangfu Fine Chemical Research Institute (Tianjin, China). Poly (ethylene oxide) (PEO 600000) was purchased from Acros Organics. PET film was purchased from Hua Zhenhua Factory Outlet Store (Shenzhen, China). Glass was purchased from South China Xiangcheng Technology. Cellulose filter paper (Whatman–Xinhua, grade 91, Hangzhou, China) was selected as the paper substrate.

Instruments. Absorption spectra were measured using a Shimadzu UV-2550 PC double-beam spectrophotometer, and dynamic data was collected using Analitik Jena Specord®210 plus UV/VIS spectrophotometer. Reflective spectroscopy of visible-light-responsive chromic materials (**VLCM**) before and after irradiation was tested via reflective mode of integrating sphere on Analitik Jena Specord®210 plus UV/VIS spectrophotometer, using barium sulfate ($BaSO_4$) as background, path length was 1 cm. Blue light source for solution test and imaging with a mask here used was Wota F25 fishing lamp (Ningbo, China). Laser automatic printing was realized by laser marking machine Xiai XYC-UV / MGL (Changchun, China). The power of the blue laser is 1.6 W and the distance of laser and **VLCM** is about 2 cm for printing. Fluorescence spectra were measured using a Shimadzu RF-5301

PC spectrophotometer. ^{1}H NMR (400, 500, 600 MHz) and ^{13}C NMR (101, 126, 151 MHz) spectra were recorded on a Bruker AVANCE400 (AVANCE500 or AVANCE600) at room temperature. LC-HRMS analysis was performed on an Agilent 1290-micro TOF-Q II mass spectrometer (electrospray ionization (ESI) source). CIE L*, a*, b* was measured by X-rite spectrodensitometer. Microscale colors of the **VLCM** were imaged in transmission, reflective and fluorescence modes using Leica DM4000 M microscope. Scanning electron microscopy (SEM) images were taken using field-emission scanning electronic microscopy (FE-SEM; SU8020, HITACHI) and field-emission environment scanning electronic microscopy (Quattro, ThermoFisher Scientific) and energy dispersive spectroscopy (EDS) images were taken using energy dispersive spectrometer (XFLASH 6/60, Bruker). Photographs were captured by using the Nikon D7100 camera and enlarged image was obtained by using microscope. The melting points were taken using an SGW X-4B microscopy melting point apparatus (Shanghai, China).

Density Functional Theory (DFT) Calculation. All structures were optimized with DFT functional B3LYP and 6-31G (d, p) basis set by Gaussian 09[S1]. The energy barriers were calculated and solvent effects with the polarizable continuum model (PCM) was considered. Computed structures were illustrated using CYLVIEW drawings.

Supplementary Synthesis Methods

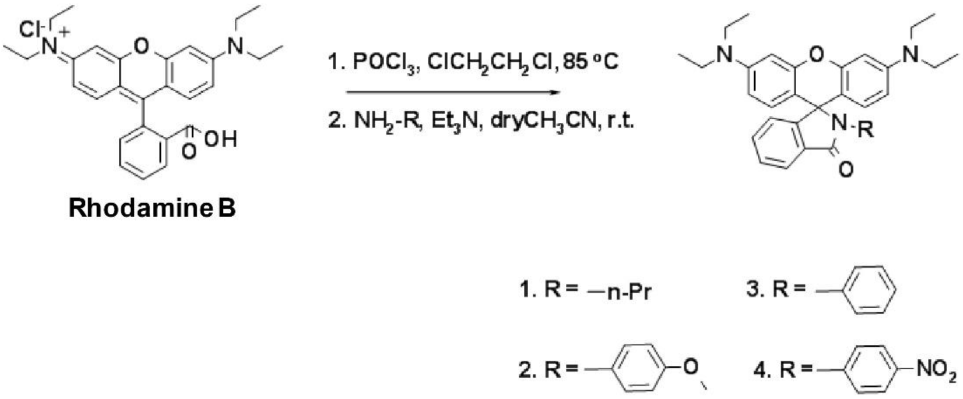

Scheme S1 Synthetic route of molecule **1**—**4**.

Preparation of compounds 1—4. Compounds **1**—**4** were prepared according to the literature[S2]. To a solution of Rhodamine B (0.67 g, 1.4 mmol) in $ClCH_2CH_2Cl$ (10 mL) at room temperature, $POCl_3$ (1.03 mL, 11.2 mmol) was added dropwise. The mixture was kept stirring at room temperature for 15 min, heated to 85 °C for 6 h and then cooled to room temperature and concentrated under vacuum to give a salt. The salt was dissolved in dry CH_3CN (10 mL), then the solution was added dropwise to a solution of desired NH_2-R (2.8 mmol) in dry CH_3CN (5 mL) containing Et_3N (10 mL). The mixture was stirred at room temperature overnight. The mixture was concentrated under vacuum.

Synthesis of 1**:** The slightly purple solid was purified by column chromatography on silica gel (Hex : EtOAc = 3 : 1) to afford **1** (589.1 mg, 87% yield). ^{1}H NMR (500 MHz, DMSO-d_6) δ 7.77 (d, J = 5.7 Hz, 1H), 7.53 – 7.46 (m, 2H), 7.04 – 6.99 (m, 1H), 6.37 – 6.29 (m, 6H), 3.33 (q, J = 6.7 Hz, 4H), 2.94 – 2.88 (m, 2H), 1.13 – 1.01 (m, 15H), 0.59 (t, J = 7.3 Hz, 3H). ^{13}C NMR (126 MHz, DMSO-d_6) δ 167.2, 153.8, 153.1, 148.8, 133.0, 131.2, 128.8, 128.7, 124.1, 122.7, 108.6, 105.7, 97.7, 64.4, 44.1, 41.9, 21.7, 12.7, 12.0. LC-HRMS (ESI) calcd. for $C_{31}H_{38}N_3O_2$ $[M + H]^+$: 484.2959, found: 484.2952.

Synthesis of 2**:** The creamy white solid was purified by column chromatography on silica gel (Hex : EtOAc = 5 : 1) to afford **2** (437.1 mg, 57% yield). ^{1}H NMR (500 MHz, DMSO-d_6) δ 7.87 (d, J = 6.4 Hz, 1H), 7.60 – 7.52 (m, 2H), 7.07 (d, J = 6.6 Hz, 1H), 6.73 (d, J = 8.9 Hz, 2H), 6.64 (d, J = 8.9 Hz, 2H), 6.56 (d, J = 8.8 Hz, 2H), 6.40 (dd, J = 8.8, 2.0 Hz, 2H), 6.26 (d, J = 2.0 Hz, 2H), 3.65 (s, 3H), 3.31 (q, J = 6.9 Hz, 8H), 1.08 (t, J = 6.9 Hz, 12H). ^{13}C NMR (126 MHz, DMSO-d_6) δ 167.1, 158.0, 153.9,152.9, 148.8, 133.6, 130.5, 129.7, 128.8, 128.5, 124.3, 123.2, 114.2, 108.4, 106.0, 97.7, 66.8, 55.5, 44.1, 12.9. LC-HRMS (ESI) calcd. for $C_{35}H_{38}N_3O_3$ $[M + H]^+$: 548.2908, found: 548.2907.

***Synthesis of 3*:** The slightly purple solid was purified by column chromatography on silica gel (Hex : EtOAc = 3 : 1) to afford **3** (311.6 mg, 43% yield). ^{1}H NMR (500 MHz, DMSO-d$_6$) δ 7.89 (d, *J* = 7.0 Hz, 1H), 7.61 – 7.53 (m, 2H), 7.17 (t, *J* = 7.6 Hz, 2H), 7.12 – 7.07 (m, 2H), 6.82 (d, *J* = 7.6 Hz, 2H), 6.57 (d, *J* = 8.8 Hz, 2H), 6.39 (d, *J* = 8.8 Hz, 2H), 6.28 (s, 2H), 3.31 (q, *J* = 6.9 Hz, 8H), 1.07 (t, *J* = 6.9 Hz, 12H). ^{13}C NMR (126 MHz, DMSO-d$_6$) δ 167.1, 158.1, 153.8, 152.8, 148.7, 133.6, 130.5, 129.7, 129.1, 128.9, 128.6, 124.3, 123.2, 114.2, 108.6, 106.0, 97.6, 66.8, 55.5, 44.1, 12.9. LC-HRMS (ESI) calcd. for $C_{34}H_{36}N_3O_2$ $[M + H]^+$: 518.2802, found: 518.2795.

***Synthesis of 4*:** The yellow solid was purified by column chromatography on silica gel (Hex : EtOAc = 5 :1) to afford **4** (362.4 mg, 46% yield). ^{1}H NMR (500 MHz, DMSO-d$_6$) δ 8.09 (d, *J* = 9.3 Hz, 2H), 7.94 (d, *J* = 7.2 Hz, 1H), 7.58 (m, 2H), 7.47 (d, *J* = 9.3 Hz, 2H), 7.05 (d, *J* = 7.5 Hz, 1H), 6.56 (d, *J* = 8.8 Hz, 2H), 6.38 (d, *J* = 2.4 Hz, 2H), 6.34 (dd, *J* = 8.8 Hz, 2.4 Hz, 2H), 3.32 – 3.26 (m, 8H), 1.07 (t, *J* = 7.0 Hz, 12H). ^{13}C NMR (126 MHz, DMSO-d$_6$) δ 168.1, 154.4, 152.3, 149.0, 144.2, 143.9, 134.7, 129.0, 128.2, 124.5, 124.1, 124.0, 123.8, 108.7, 105.8, 97.9, 67.0, 44.0, 12.9. LC-HRMS (ESI) calcd. for $C_{34}H_{35}N_4O_4$ $[M + H]^+$: 563.2653, found: 563.2649.

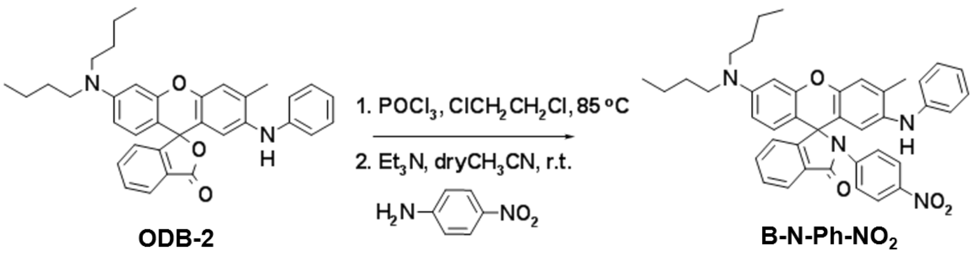

Scheme S2 Synthetic route of molecule **B-N-Ph-NO$_2$**.

***Synthesis of B-N-Ph-NO$_2$*:** Its synthetic process was simliar to **4**. ODB-2 (1.14 g, 2.0 mmol) and $POCl_3$ (1.3 mL, 14 mmol) were dissolved in 12 mL $ClCH_2CH_2Cl$, and the mixture was stirred at 85 °C for 6 h. Then the solvent was removed and stirred at room temperature with p-nitroaniline (5.0 mmol,) CH_3CN (15 mL) containing Et_3N (4.2 mL) overnight. **B-N-Ph-NO$_2$** was purified by column chromatography on silica gel (Hex : EtOAc = 3 : 1) (705.6 mg, 54% yield). Mp: 107.2 °C-107.7 °C. ^{1}H NMR (400 MHz, $CDCl_3$) δ 8.04 (d, *J* = 8.9 Hz, 2H), 7.95 (d, *J* = 7.3 Hz, 1H), 7.64-7.43 (m, 4H), 7.12 (d, *J* = 7.4 Hz, 1H), 7.08 – 7.00 (m, 3H), 6.75 (t, *J* = 7.3 Hz, 1H), 6.63 (d, *J* = 5.3 Hz, 2H), 6.42 – 6.28 (m, 4H), 5.15 (s, 1H), 3.25(t, *J* = 7.4 Hz, 4H), 2.19 (s, 3H), 1.65 – 1.53 (m, 4H), 1.40 – 1.31 (m, 4H), 0.97 (t, *J* = 7.2 Hz, 6H). ^{13}C NMR (126 MHz, $CDCl_3$) δ 168.2, 153.4, 153.1, 149.0, 147.2, 143.9, 138.1, 132.5, 131.2, 129.3, 129.1, 128.3, 123.7, 123.0, 121.1, 120.9, 120.3, 118.3, 117.7, 116.2, 108.4, 104.8,

97.7, 65.0, 44.5, 42.5, 21.9, 12.7, 11.7. LC-HRMS (ESI) calcd. for $C_{41}H_{41}N_4O_4$ $[M + H]^+$: 653.3122, found: 653.3128.

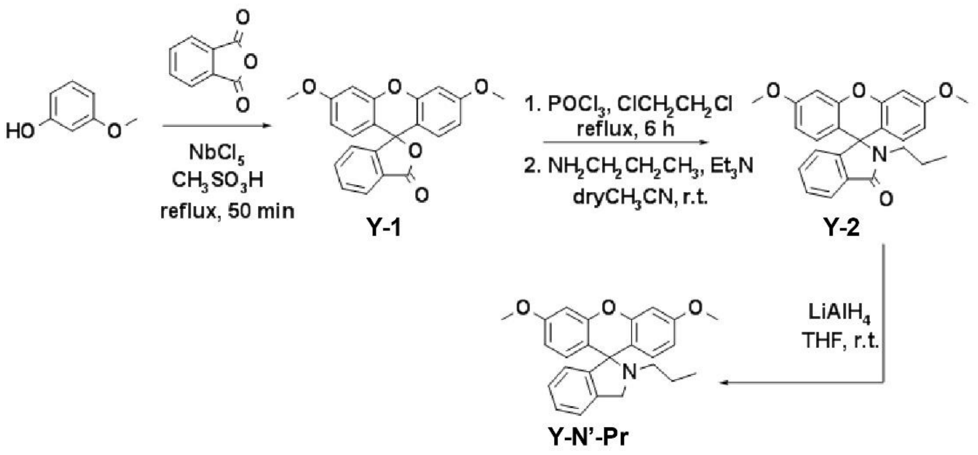

Scheme S3 Synthetic route of molecule **Y-N'-Pr**.

Synthesis of (S3-(Y-1)) [S3]**:** 3-Methoxyphenol (5.2 mL, 45 mmol) and phthalic anhydride (4.0 g, 25 mmol) were dissolved in a solution of $NbCl_5$ (1.8 g, 6.5 mmol) in CH_3SO_3H (250 mL). The reaction was performed in a nitrogen atmosphere with heating at 98 ^oC for 50 min. The reaction was poured into iced distilled H_2O and the precipitate was filtered under reduced pressure and washed with ice H_2O. The solid was purified by column chromatography on silica gel (Hex : EtOAc = 10 : 1) to afford **Y-1** (2.53 g, 28% yield). ^1H NMR (400 MHz, $CDCl_3$) δ 8.02 (d, *J* = 7.2 Hz, 1H), 7.69 – 7.59 (m, 2H), 7.16 (d, *J* = 7.5 Hz, 1H), 6.78 (d, *J* = 2.4 Hz, 2H), 6.70 (d, *J* = 8.8 Hz, 2H), 6.62 (dd, *J* = 8.8, 2.4 Hz, 2H), 3.84 (s, 6H).

Synthesis of (S3-(Y-2)): Its synthetic process was simliar to **1**. **Y-1** (1.08 g, 3.0 mmol) and $POCl_3$ (2.25 mL, 24 mmol) were dissolved in 12 mL $ClCH_2CH_2Cl$, and the mixture were stirred at 85 ^oC for 6 h. Then the solvent was removed and stirred at room temperature with propylamine (3.6 mmol), CH_3CN (10 mL) containing Et_3N (4.9 mL) overnight. **Y-2** was purified by column chromatography on silica gel (Hex : EtOAc = 20 : 1) (505.3 mg, 42% yield). ^1H NMR (400 MHz, $CDCl_3$) δ 7.93 (dd, *J* = 6.0, 2.0 Hz, 1H), 7.49 – 7.41 (m, 2H), 7.01 – 6.98 (m, 1H), 6.71 (d, *J* = 2.3 Hz, 2H), 6.57 (d, *J* = 8.7 Hz, 2H), 6.54 (dd, *J* = 8.8, 2.3 Hz), 3.83 (s, 6H), 3.08 – 3.03 (m, 2H), 1.17 – 1.07 (m, 2H), 0.66 (t, *J* = 7.4 Hz, 3H).

Synthesis of Y-N'-Pr**:** **Y-2** (350 mg, 0.87 mmol) was dissolved in anhydrous THF (5 ml). The solution was added $LiAlH_4$ (330 mg, 8.7 mmol). The mixture was stirred under argon overnight. Extracted by EtOAc and H_2O, the organic layer was collected and concentrated under vacuum. The yellow solution was purified by column chromatography on silica gel (Hex : EtOAc = 20 : 1) to afford

Y-N'-Pr (320.8 mg, 95% yield). ^{1}H NMR (400 MHz, DMSO-d$_6$) δ 7.39 (d, *J* = 7.4 Hz, 1H), 7.26 (t, *J* = 7.3 Hz, 1H), 7.15 (t, *J* = 7.4 Hz, 1H), 6.72-6.66 (m, 5H), 6.60 (d, *J* = 8.6 Hz, 2H), 4.16 (s, 2H), 3.75 (s, 6H), 2.06 (t, *J* = 6.9 Hz, 2H), 1.26 (q, *J* = 7.1 Hz, 2H), 0.58 (t, *J* = 7.2 Hz, 3H).^{13}C NMR (101 MHz, DMSO-d$_6$) δ 159.1, 151.5, 149.1, 138.5, 130.5, 127.6, 127.1, 123.7, 122.3, 116.3, 110.5, 99.9, 67.3, 55.6, 55.2, 49.7, 21.4, 11.6. LC-HRMS (ESI) calcd. for $C_{25}H_{26}NO_3$ [M + H]$^+$: 388.1914, found: 388.1907.

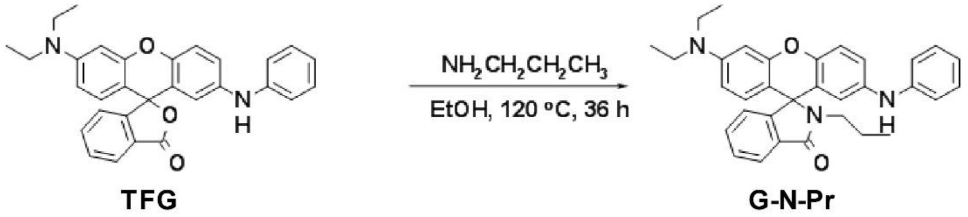

Scheme S4 Synthetic route of molecule **G-N-Pr**.

***Synthesis of G-N-Pr*:** TFG (1.0 g, 2.16 mmol) and $NH_2CH_2CH_2CH_3$ (0.35 mL, 4.32 mmol) were added to a Schlenk tube flask containing EtOH (10 mL). The mixture was stirred at 120 ^{o}C for 36 h. The mixture was purified by silica gel column chromatography (CH_2Cl_2 : EtOAc = 15 : 1) to afford **G-N-Pr** (927 mg, 85% yield). Mp: 211.5 ^{o}C -212.0 ^{o}C. ^{1}H NMR (400 MHz, DMSO-*d*$_6$) δ 8.01 (s, 1H), 7.80 – 7.77 (m, 1H), 7.53 (t, *J* = 6.3 Hz, 2H), 7.18 (d, *J* = 9.0 Hz, 1H), 7.15 – 7.04 (m, 4H), 6.77 (d, *J* = 8.2 Hz, 2H), 6.71 (t, *J* = 7.4 Hz, 1H), 6.42 – 6.37 (m, 2H), 6.36 – 6.32 (m, 1H), 6.25 (d, *J* = 2.3 Hz, 1H), 3.35-3.30 (m, 3H), 3.00 – 2.94 (m, 2H), 1.08 (t, J = 6.9 Hz, 9H), 0.61 (t, *J* = 7.4 Hz, 3H). ^{13}C NMR (126 MHz, $CDCl_3$) δ 168.2, 153.4, 153.1, 149.0, 147.2, 143.9, 138.1, 132.5, 131.2, 129.3, 129.1, 128.3, 123.7, 123.0, 121.1, 120.9，120.3, 118.3, 117.7, 116.2, 108.4, 104.8，97.7, 65.0, 44.5, 42.5, 21.9, 12.7, 11.9. LC-HRMS (ESI) calcd. for $C_{33}H_{34}N_3O_2$ [M + H]$^+$: 504.2646, found: 504.2656.

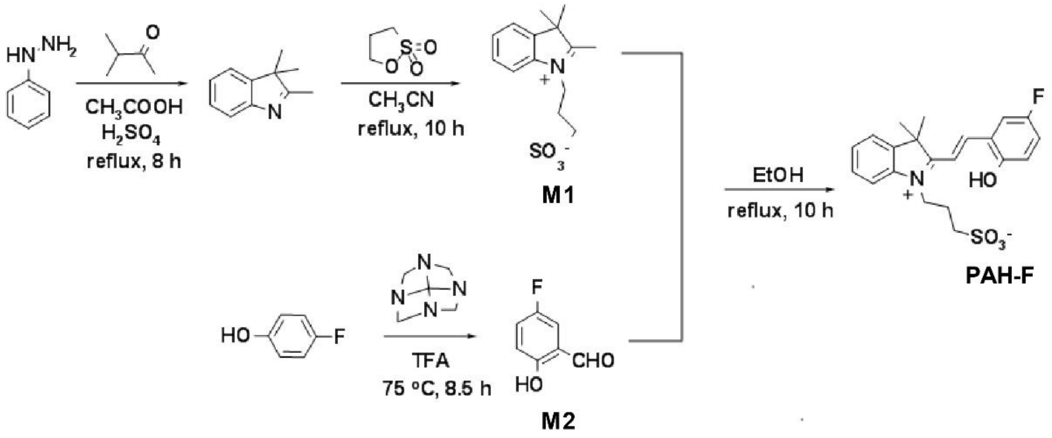

Scheme S5 Synthetic route of molecule **PAH-F**.

Synthesis of 2,3,3-trimethyl-1-(3-sulfonatepropyl)-3H-indolium (S5-(M1)): Its was synthesized following a literature procedure[S4]. (85% yield). ^{1}H NMR (500 MHz, DMSO-d_6) δ 8.05 (d, *J* = 7.0 Hz, 1H), 7.82 (d, *J* = 6.5 Hz 1H), 7.66 – 7.56 (m, 2H), 4.66 (t, *J* = 7.5 Hz, 2H), 2.83 (s, 3H), 2.62 (t, *J* = 6.5 Hz, 2H), 2.18 – 2.13 (m, 2H), 1.53 (s, 6H). ^{13}C NMR (126 MHz, DMSO-d_6) δ 196.6, 142.0, 141.2, 129.4, 129.0, 123.4, 115.5, 54.2, 47.4, 46.7, 23.8, 22.1, 13.9.

Synthesis of 5-fluoro-2-hydroxybenzaldehyde (S5-(M2)): Its was synthesized following a literature procedure[S5]. (20% yield). ^{1}H NMR (500 MHz, DMSO-d_6) δ 10.69 (s, 1H), 10.25 (s, 1H), 7.42 – 7.34 (m, 2H), 7.03 (m, 1H). ^{13}C NMR (126 MHz, DMSO-d_6) δ 189.92, 157.21, 156.20 (d, *J* = 237.20 Hz), 123.50 (d, *J* = 24.00 Hz), 122.64 (d, *J* = 4.50 Hz), 119.07 (d, *J* = 5.51 Hz), 113.10 (d, *J* = 22.81 Hz).

Synthesis of PAH-F: 2,3,3-trimethyl-1-(3-sulfonatepropyl)-3H-indolium (710 mg, 0.025 mol) and 5-fluoro-2-hydroxybenzaldehyde (530 mg, 0.038 mol) were added into EtOH (10 mL). The mixture was allowed to reflux overnight. The orange solid was obtained by filtration and washed with EtOAc to afford PAH-F (879.2 mg, 86% yield). ^{1}H NMR (500 MHz, DMSO-d_6) δ 11.00 (s, 1H), 8.51 (d, *J* = 16.4 Hz, 1H), 8.20 (dd, *J* = 9.8, 2.9 Hz, 1H), 8.05 (d, *J* = 7.2 Hz, 1H), 7.91 – 7.86 (m, 2H), 7.67 – 7.60 (m, 2H), 7.34 (td, *J* = 8.6, 3.1 Hz, 1H), 7.04 (dd, *J* = 9.0, 4.7 Hz, 1H), 4.84 (t, *J* = 7.7 Hz, 2H), 2.65 (t, *J* = 6.5 Hz, 2H), 2.19 (m, 2H), 1.77 (s, 6H). ^{13}C NMR (151 MHz, DMSO-d_6) δ = 181.69, 156.66 (d, *J* = 236.88 Hz), 155.35, 147.12, 143.62, 140.87, 129.37, (d, *J* = 23.94 Hz), 123.00, 122.60 (d, *J* = 23.94 Hz), 121.88 (d, *J* = 7.56 Hz), 117.97 (d, *J* = 6.30 Hz), 115.28, 114.34 (d, *J* = 23.94 Hz), 112.59, 52.04, 47.28, 45.69, 25.30, 24.68. LC-HRMS (ESI) calcd. for $C_{21}H_{23}FNO_4S$ $[M + H]^+$: 404.1326, found: 404.1320.

Preparation of LCM. The **LCM** was prepared in a layer-by-layer manner similar to our previous work[S6, S7]. The filter paper was firstly soaked into a 20 wt% PEG20000 aqueous solution and then dried by heating at 80 °C. After that, **LCM** was obtained by soaking the treated filter into MeOH-CH_2Cl_2 mixture solution within acidochromic dyes (*i.e.*, **1**—**4**) (2 mM), PAH-F (2.4 mM) and 10 wt% PEG20000 mixture.

General test method for the prontonation kinetics of 1—4 with PAH-F. Unless otherwise specified, all the kinetic data for the prontonation of 1—4 by PAH-F was obtained according to the following method: PAH-F in MeOH was firstly added to the MeOH solution of **1**—**4**. The mixture of PAH-F and **1**—**4** was then irradiated by a blue LED light till the fully conversion of PAH-F to SP-F (set

as time = 0, the initial time of kinetics test), which could be visualized as the mixture turned to be transparent from yellow. The mixture was kept irradiating, illustrating that the photoacid was in its SP-F form during the measurement. While its spectra was measured at set time intervals and the kinetics data was obtained by measuring the maximum absorption changes of the protonated **1—4** with time.

General test method for the prontonation kinetics of 1—4 with CH_3SO_3H. CH_3SO_3H in MeOH was added to the MeOH solution of **1—4** at time = 0 s (the initial time of kinetics test). The kinetics data was obtained by measuring the maximum absorption changes of the protonated **1—4** with time.

Supplementary Figures

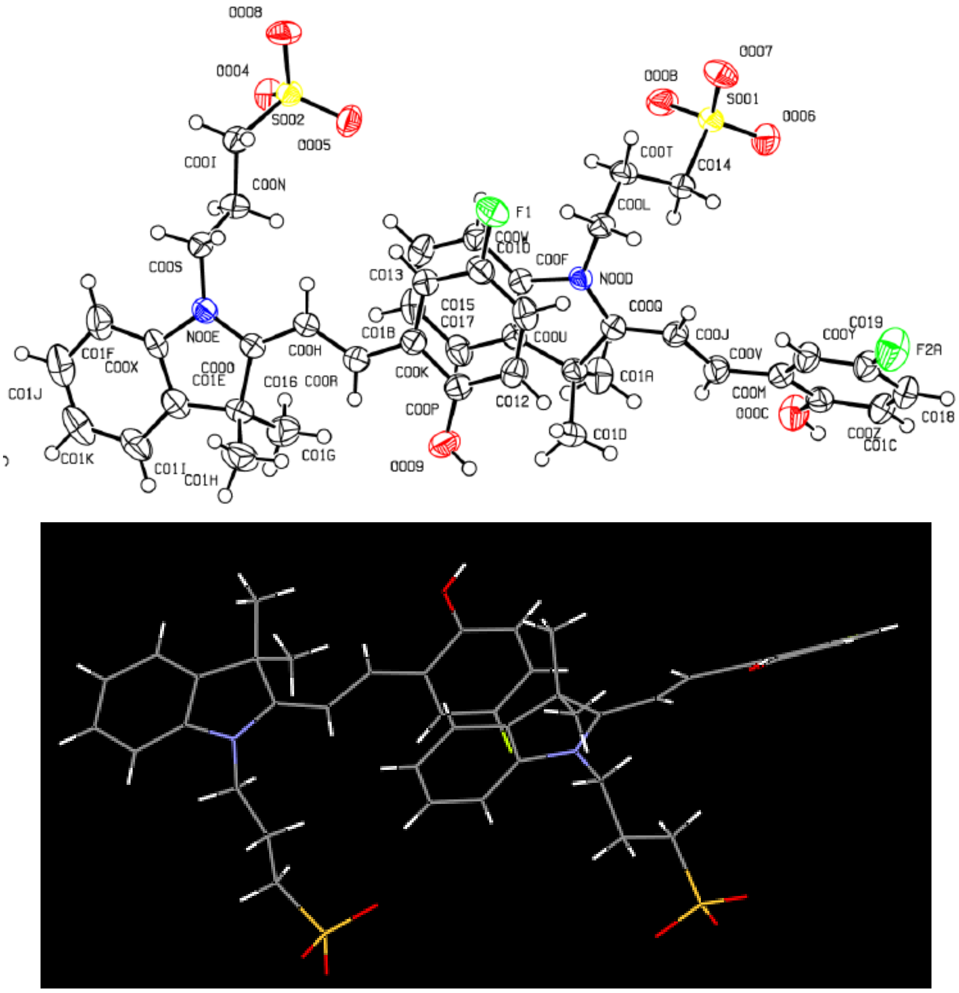

Fig. S1 X-ray single crystal diffraction of PAH-F.

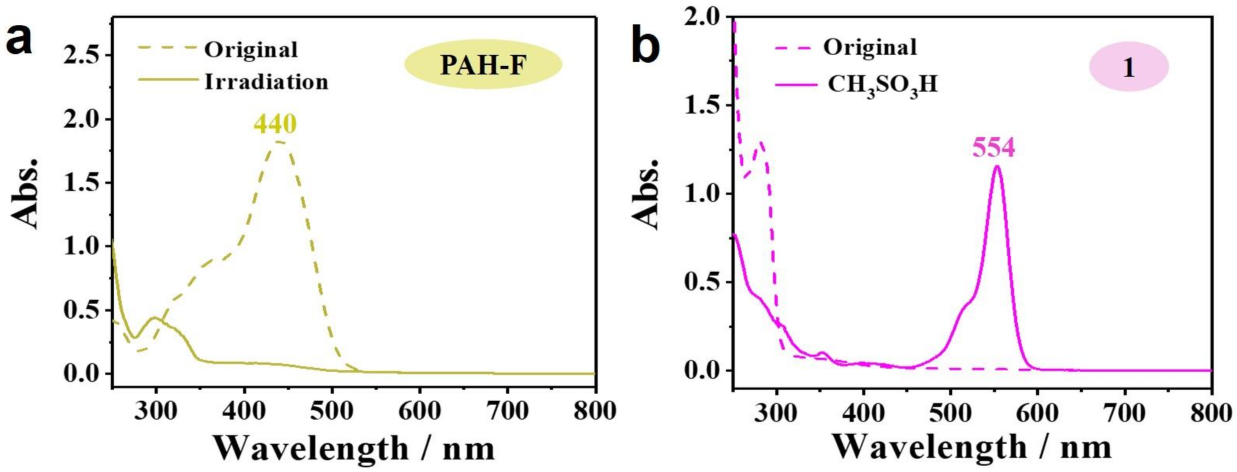

Fig. S2 UV-vis absorption spectra of (**a**) PAH-F (0.5 mM, MeOH) before and after blue light irradiation, (**b**) **1** (0.025 mM, MeOH) before and after protonation, and **1** was completely protonated after adding 10 equivalents CH_3SO_3H and equilibrating for 17 hours.

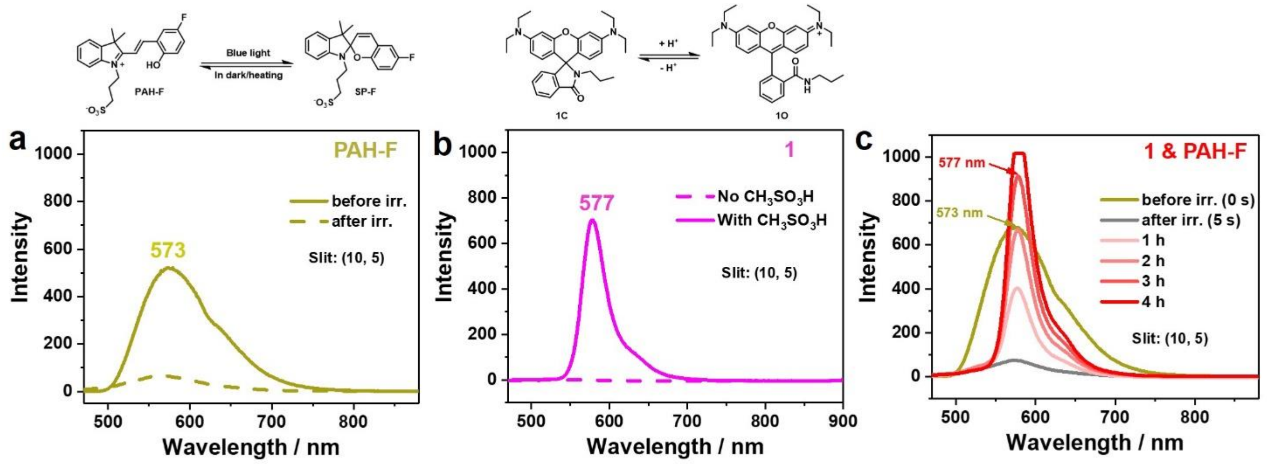

Fig. S3 Fluorescence spectra of (**a**) PAH-F (0.5 mM, MeOH) before and after blue light irradiation, (**b**) **1** (0.025 mM, MeOH) before and after protonation and (**c**) **1** & PAH-F (10 equivalents) in MeOH solution at ambient temperature, recorded with varying irradiation time for 0 s or 5 s to 4 h, as well as structure illustration of PAF-F photoisomerization and **1** protonation process (top). (λ_{ex} = 450 nm, slit width (10, 5)).

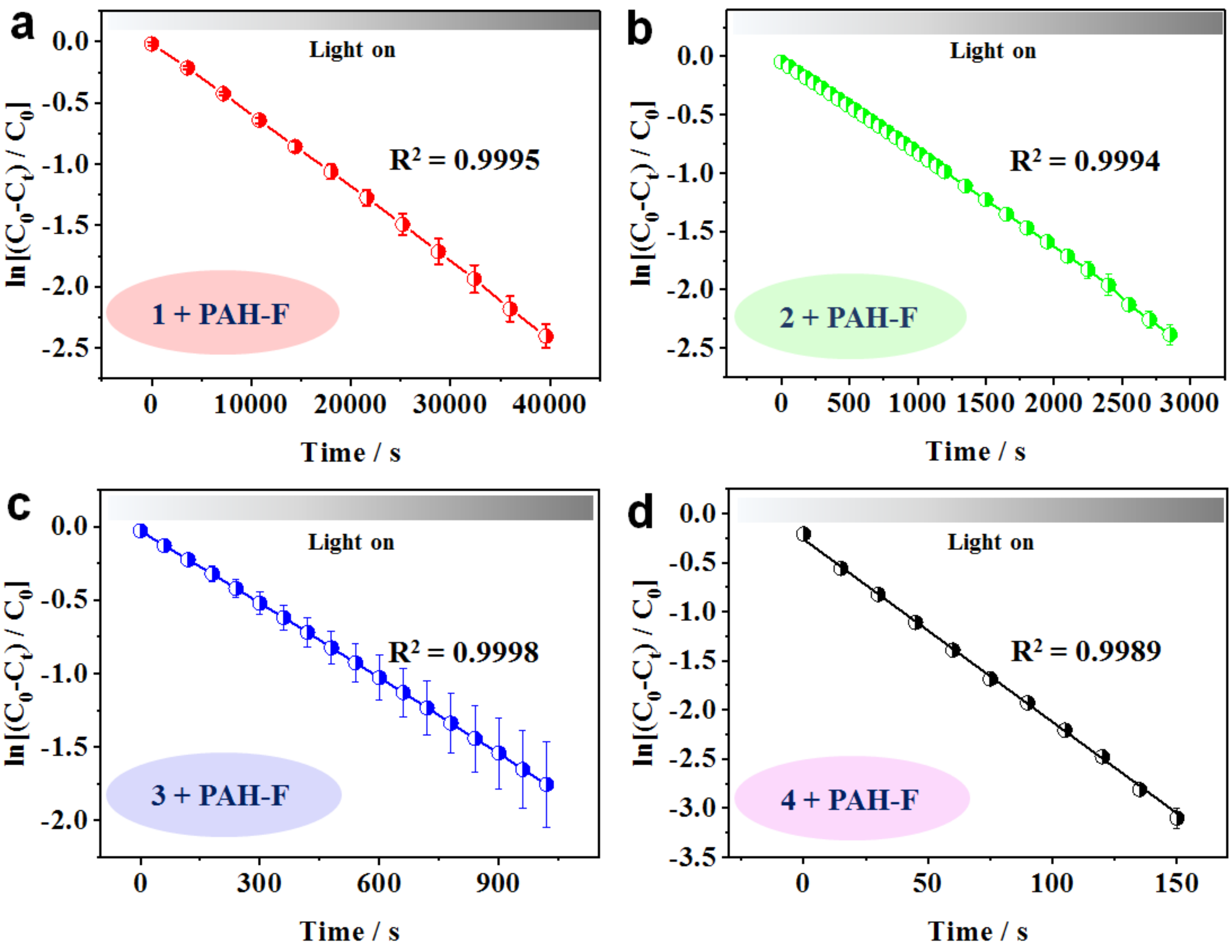

Fig. S4 First order kinetic curve of protonation of (**a**) **1**, (**b**) **2**, (**c**) **3** and (**d**) **4** (0.025 mM, MeOH) by using 10 equivalent PAH-F at 25 °C, respectively. During the whole test process, the solutions were kept irradiating by a blue light.

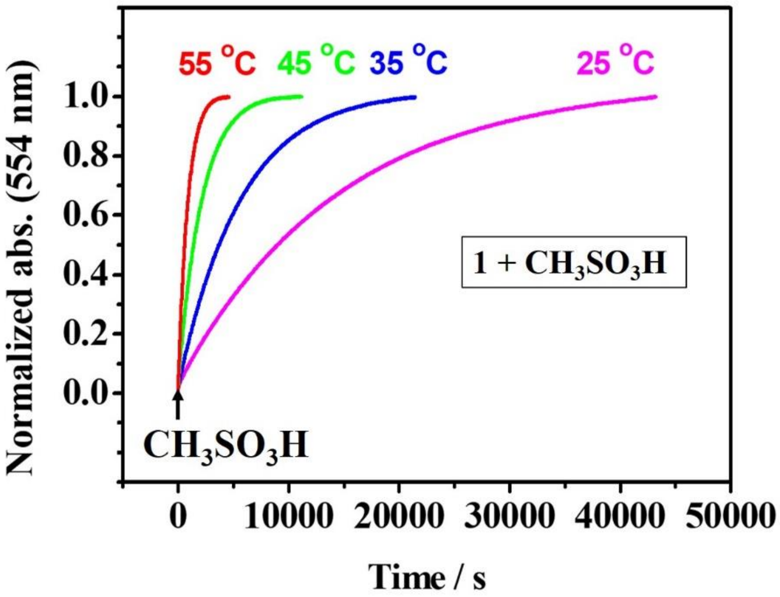

Fig. S5 Normalized kinetic data of **1** (0.025 mM, MeOH) at different temperatures treated with 10 equivalent CH_3SO_3H.

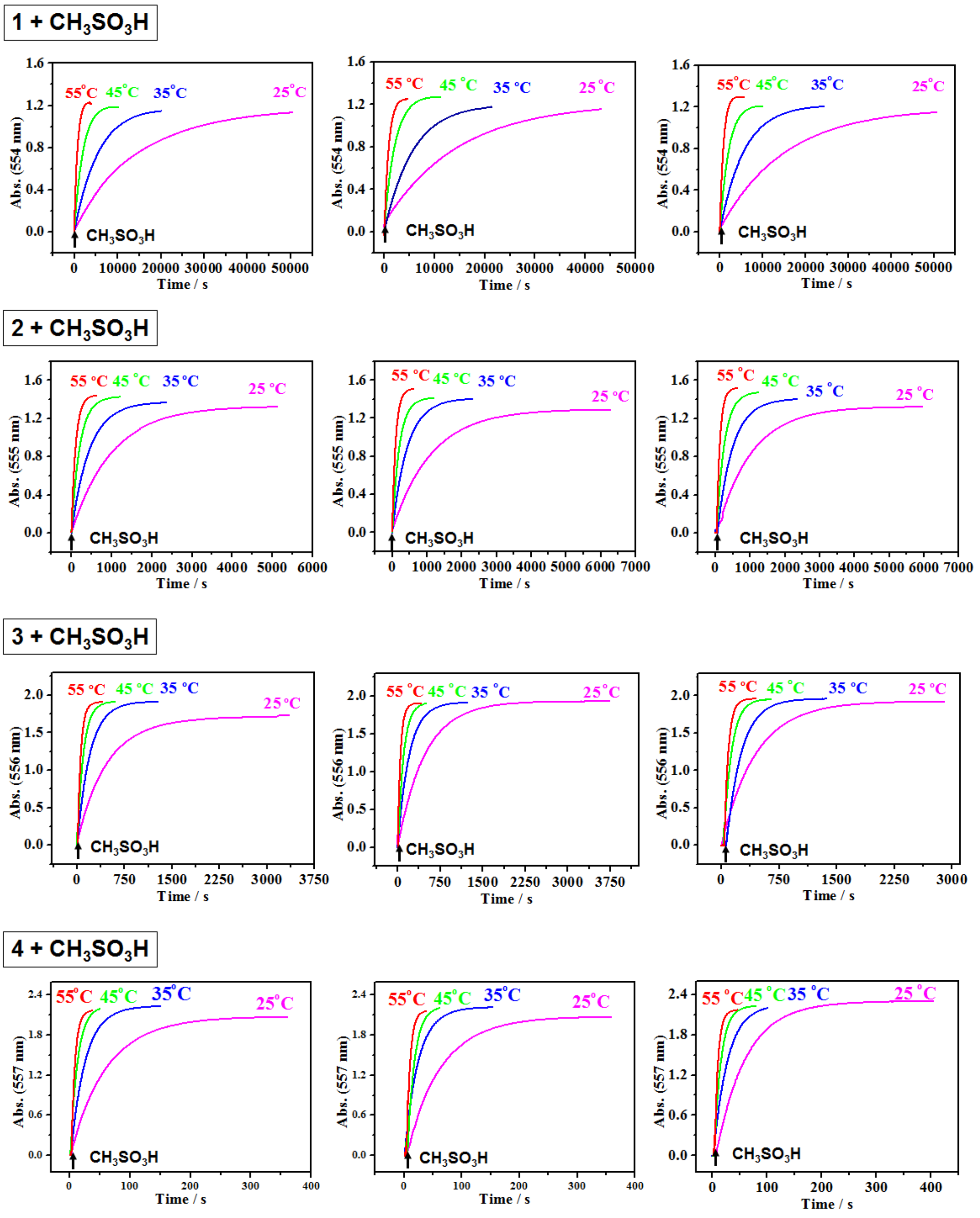

Fig. S6 Three parallel experiments of kinetic data of **1**—**4** (0.025 mM, MeOH) under the treatment of 10 equivalents CH_3SO_3H at different temperatures.

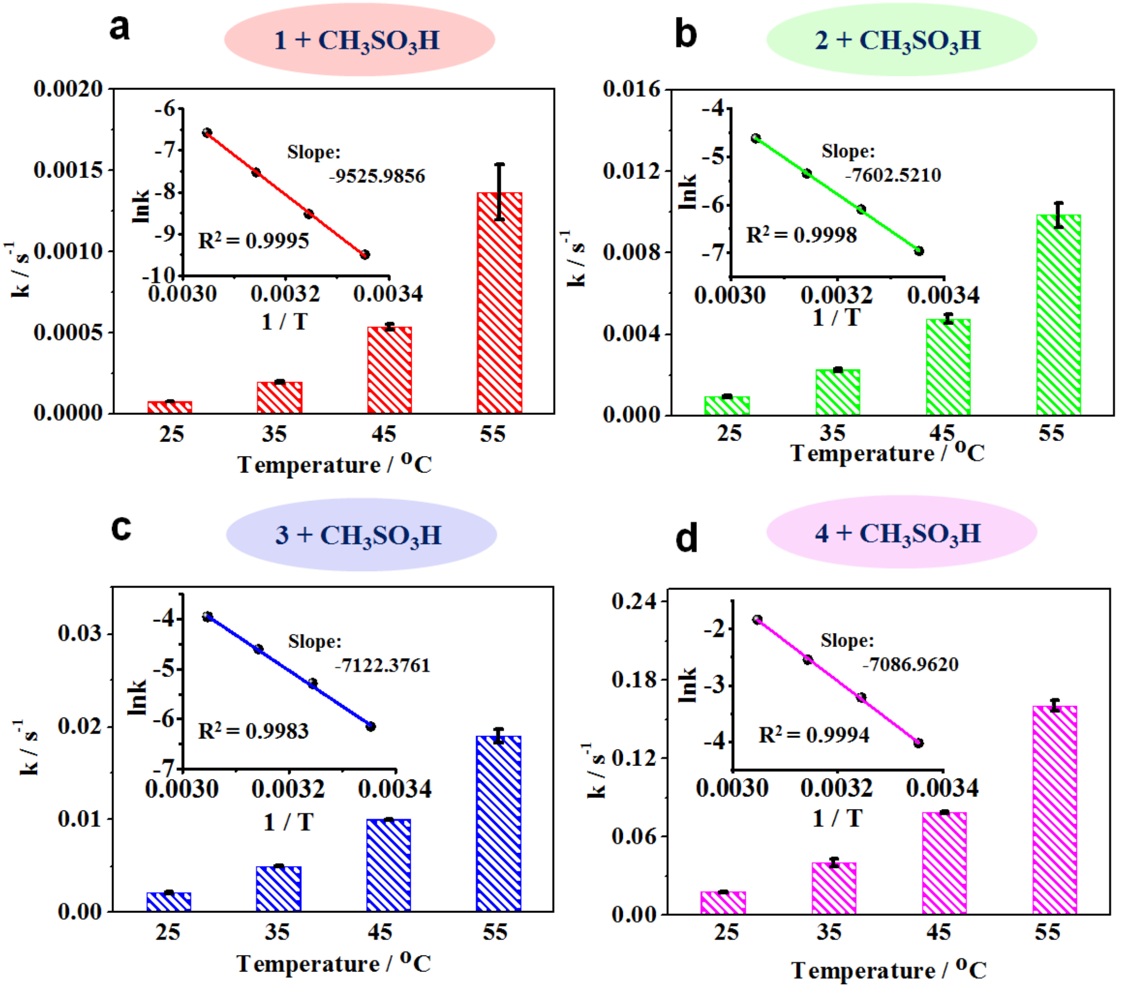

Fig. S7 The protonation rates of (**a**) **1**, (**b**) **2,** (**c**) **3** and (**d**) **4** (0.025 mM, MeOH) by using 10 equivalent CH_3SO_3H at different temperatures. Inserts show the liner relation between lnk and 1/T of **1**—**4**.

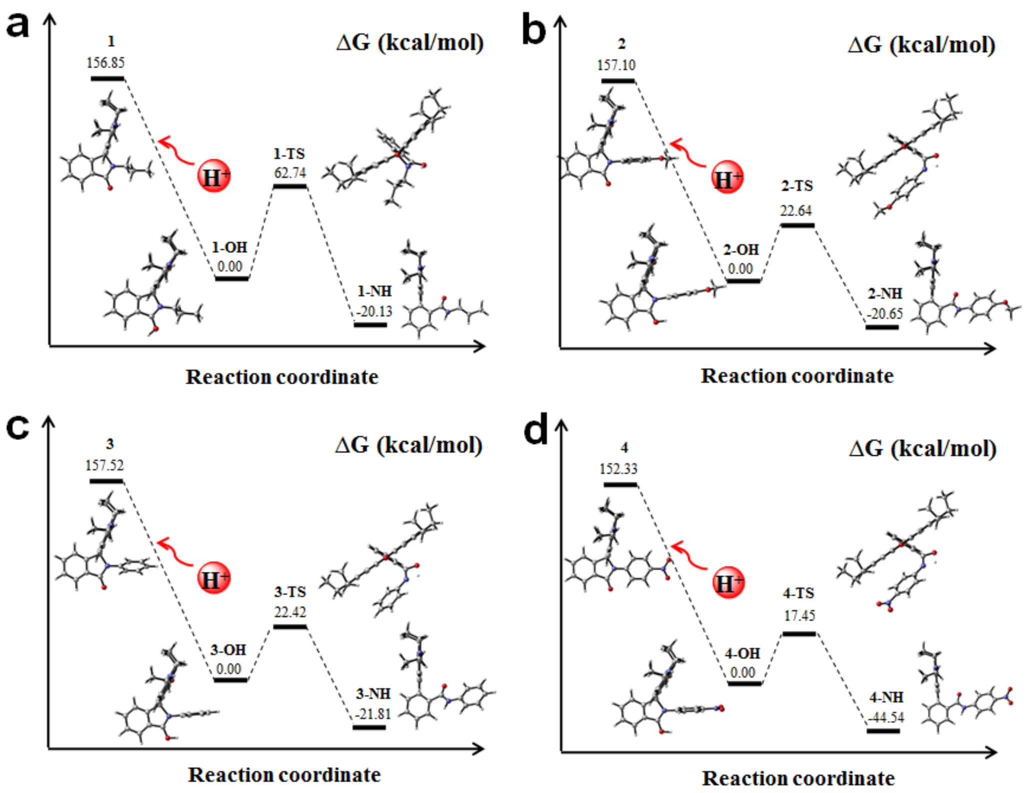

Fig. S8 Free energy curves (in kcal / mol) for protonation process of (**a**) **1**, (**b**) **2**, (**c**) **3** and (**d**) **4**.

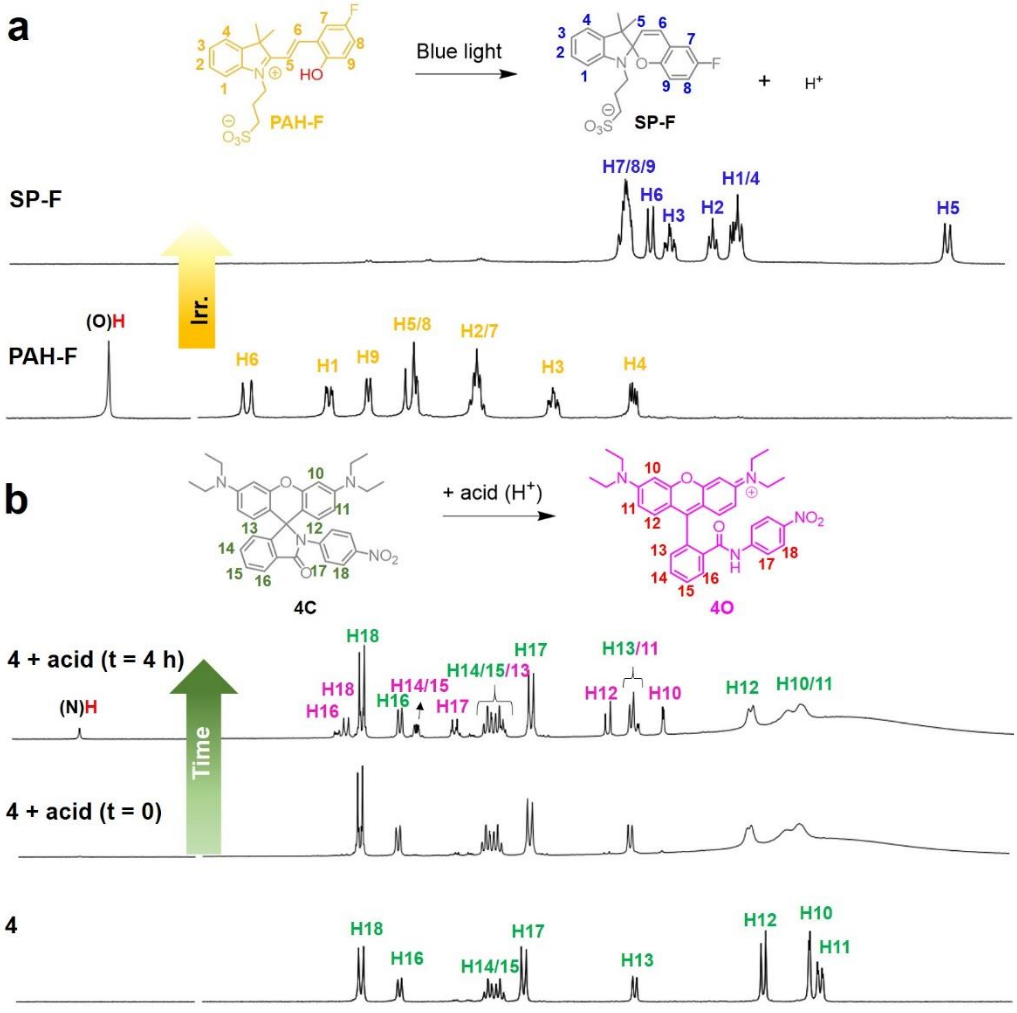

Fig. S9 ^{1}H NMR spectra (500 MHz, DMSO-d_6) of (**a**) PAH-F before and after irradiation, (**b**) **4** before and after treated with 10 equivalent CF_3COOD.

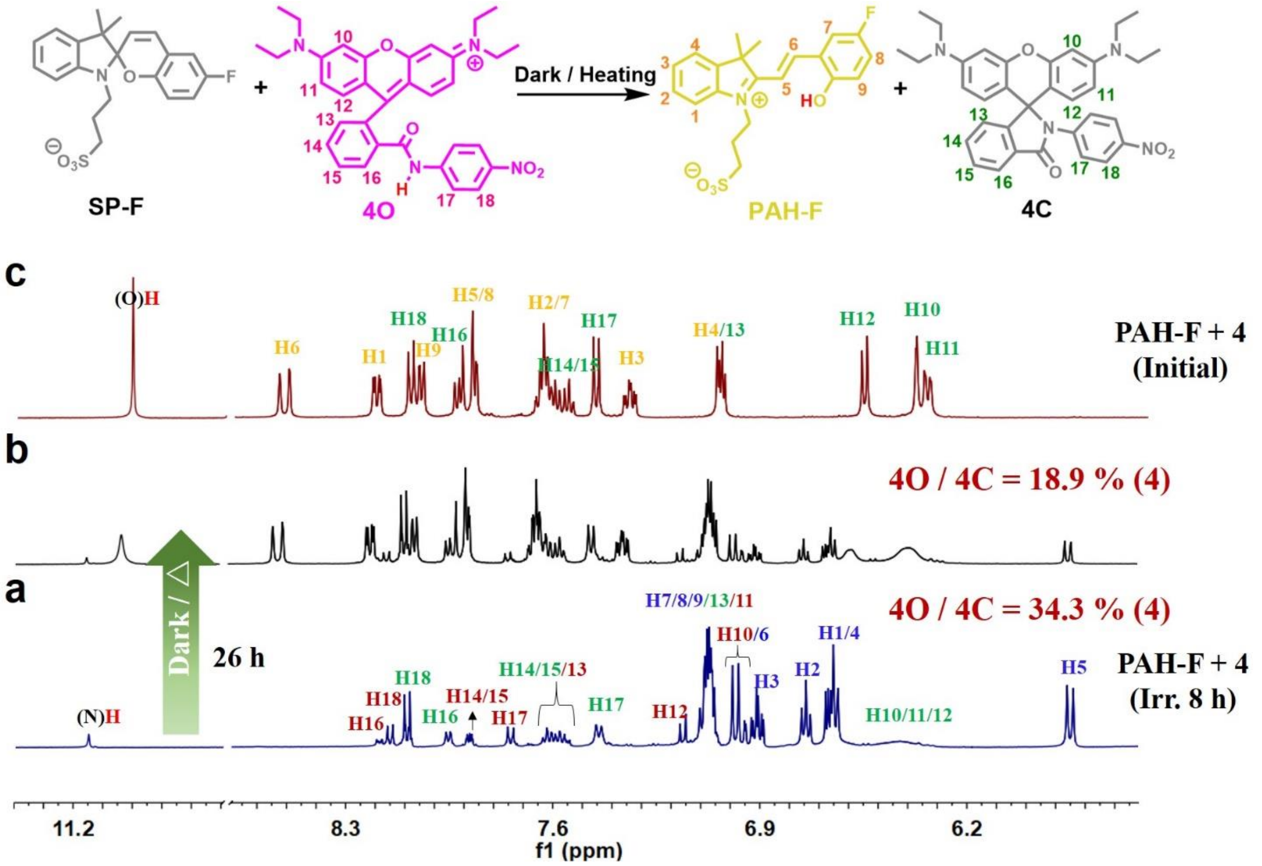

Fig. S10 The ^{1}H NMR spectra (500 MHz, DMSO-d_6) of (**a**) mixture of **4** and 2 equivalent PAH-F after continuously blue light irradiation for 8 h, (**b**) the irradiated mixture of (**a**) kept in dark at room temperature for 20 h and heated at 60 ^{o}C for about 6 h, and (**c**) the initial mixture of PAH-F & **4**.

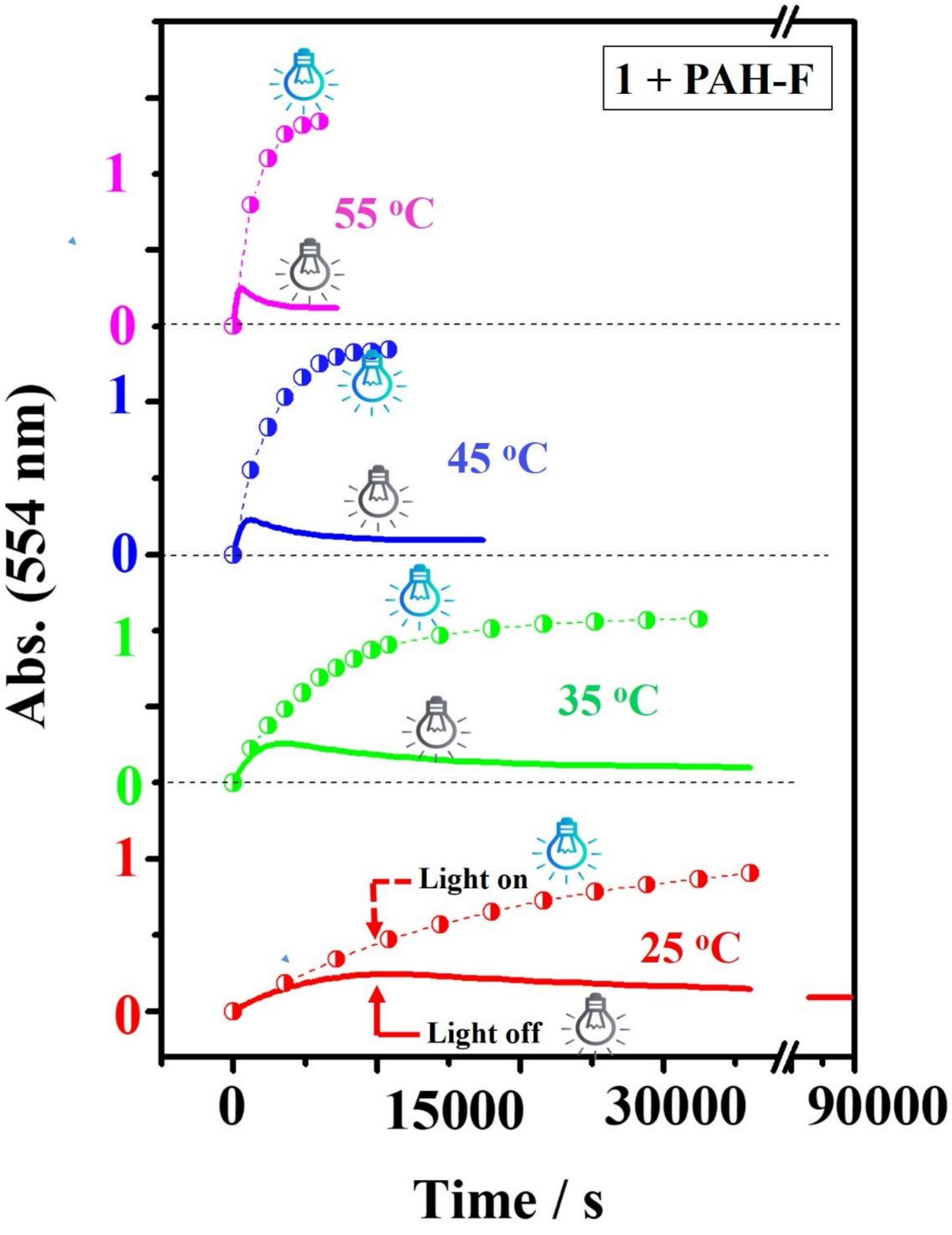

Fig. S11 UV-vis absorbance changes at 554 nm for **1** (0.025 mM, MeOH) treated with 10 equivalent PAH-F (MeOH) at different temperatures with time, recorded with different conditions for sustaining blue light irradiation (dot line) or in dark immediately after a brief blue light irradiation (solid line).

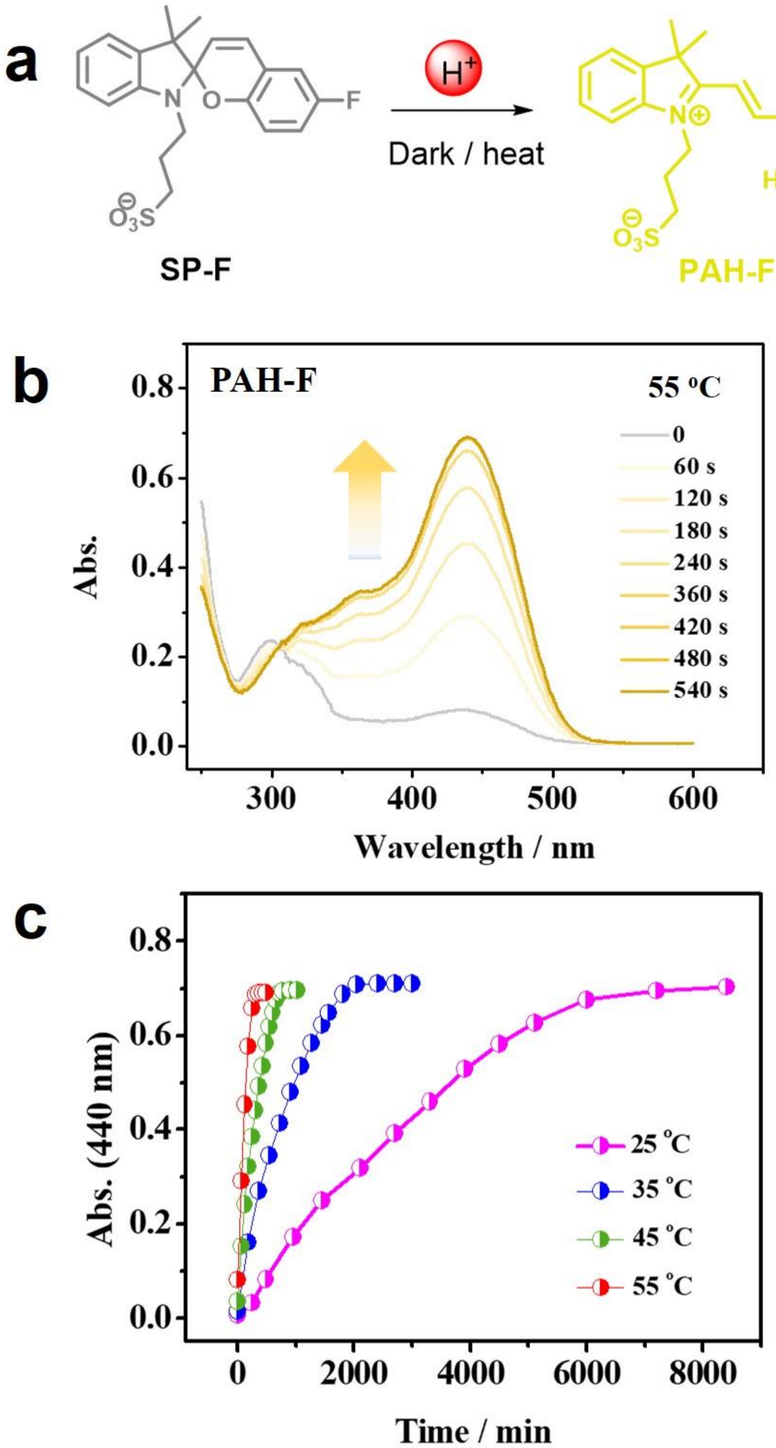

Fig. S12 (**a**) Sturcture illustration of the relaxation process from SP-F to PAH-F. (**b**) UV-vis spectra of PAH-F (0.025 mM, MeOH) during relaxation process at 55 ^{o}C, recorded with different time. (**c**) UV-vis absorbance changes at 440 nm for PAH-F (0.025 mM, MeOH) at different temperatures during relaxation process. During the whole relaxation process, the solutions were kept in dark.

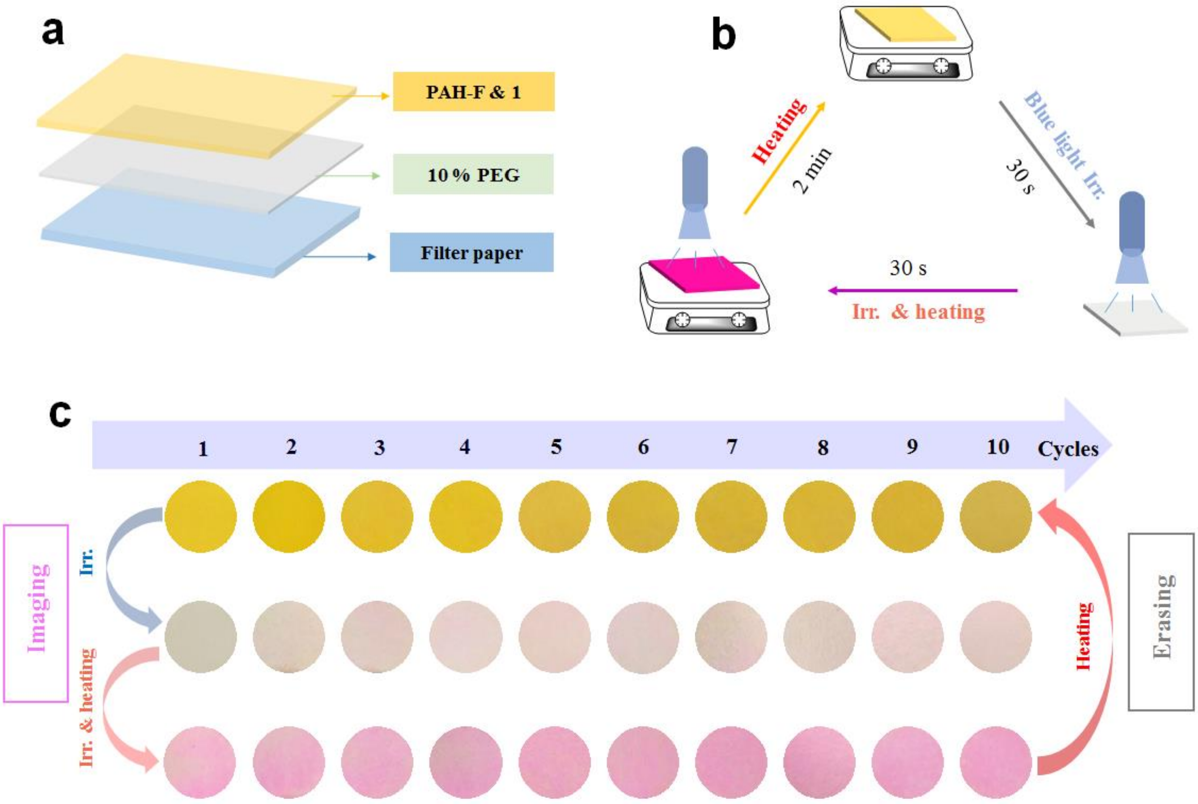

Fig. S13 (**a**) Scheme of PEG integrated three-layer structure for the rewritable media based on PAH-F (2.4 mM) and **1** (2 mM). (**b**) Schematic illustrations of reversible printing on the rewritable media. (**c**) Photographs of this media during 10 consecutive print–erase rounds.

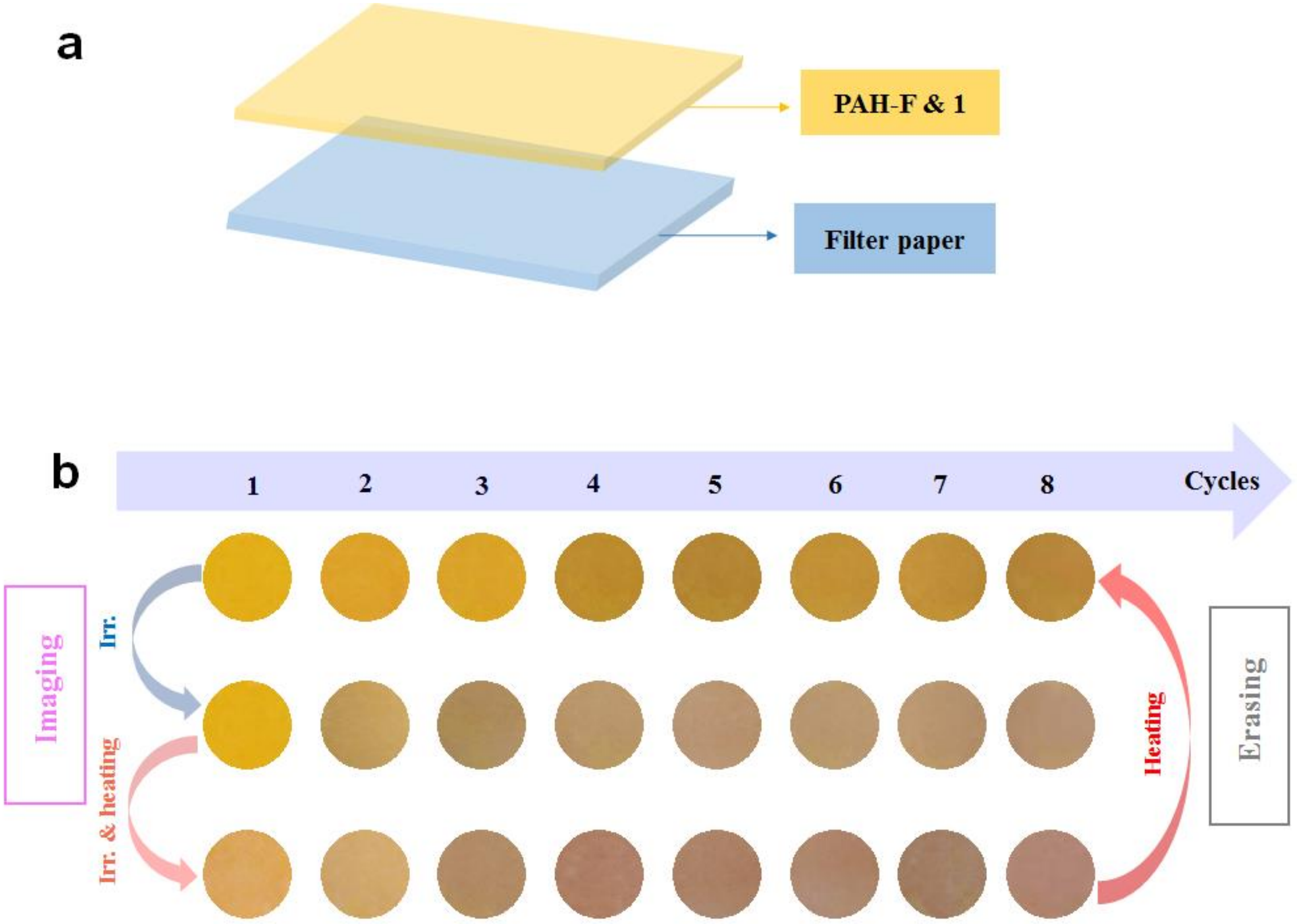

Fig. S14 (**a**) Scheme of two-layer structure for the paper based on PAH-F (2.4 mM) and **1** (2 mM) without PEG. (**b**) Photographs of this paper during 8 consecutive print–erase rounds.

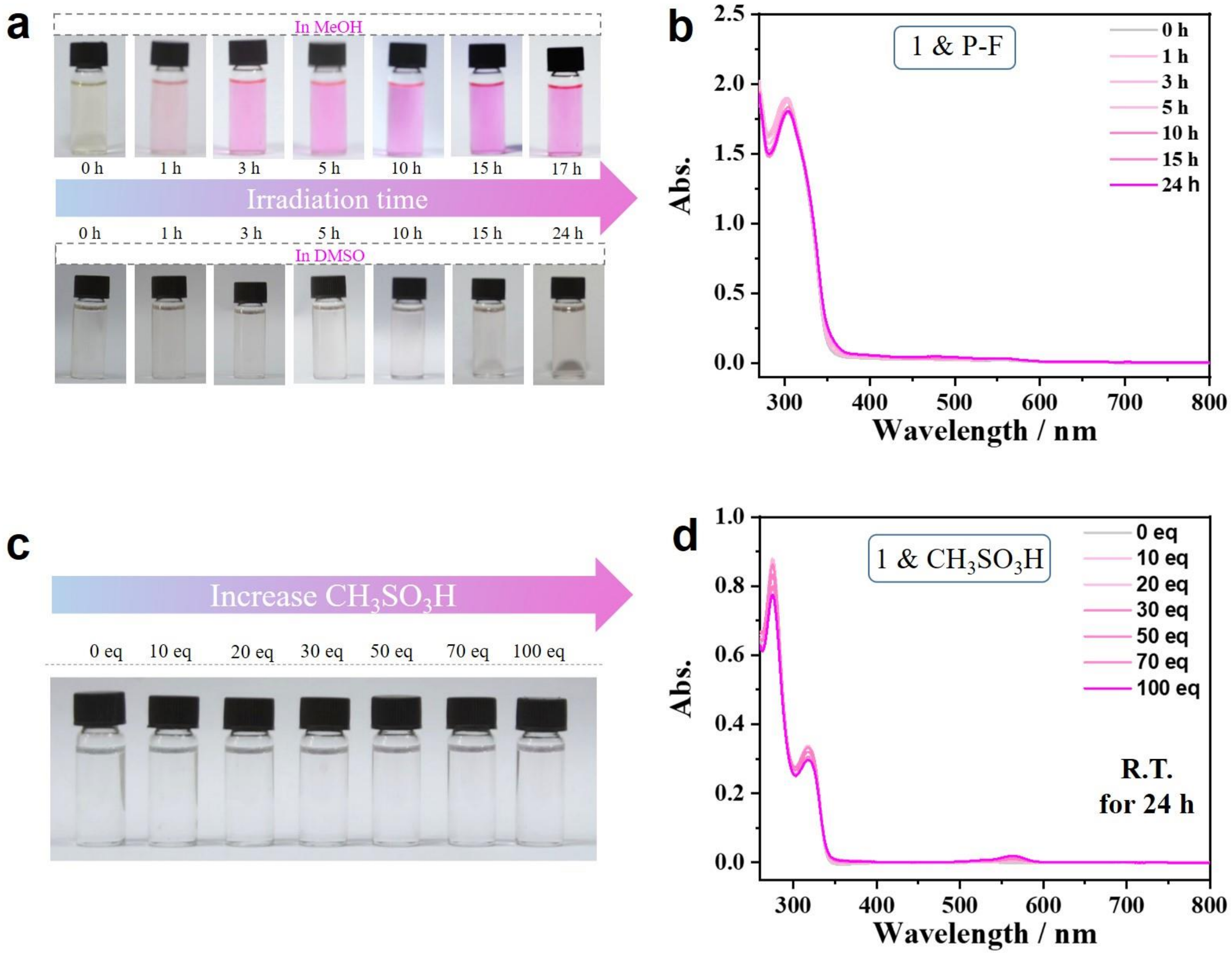

Fig. S15 (**a**) Photographs of color changes with time of the mixture (**1** and PAH-F) kept irradiating in MeOH and DMSO solution. (**b**) UV-vis spectra changes of the mixture (**1** and PAH-F) kept irradiating in DMSO solution. (**c**) Photographs of **1** (0.025 mM, DMSO) treated with 0-100 equivalent CH_3SO_3H after equilibrating for 24 h. (**d**) UV-vis spectra of **1** (0.025 mM, DMSO) treated with 0-100 equivalent CH_3SO_3H after equilibrating for 24 h.

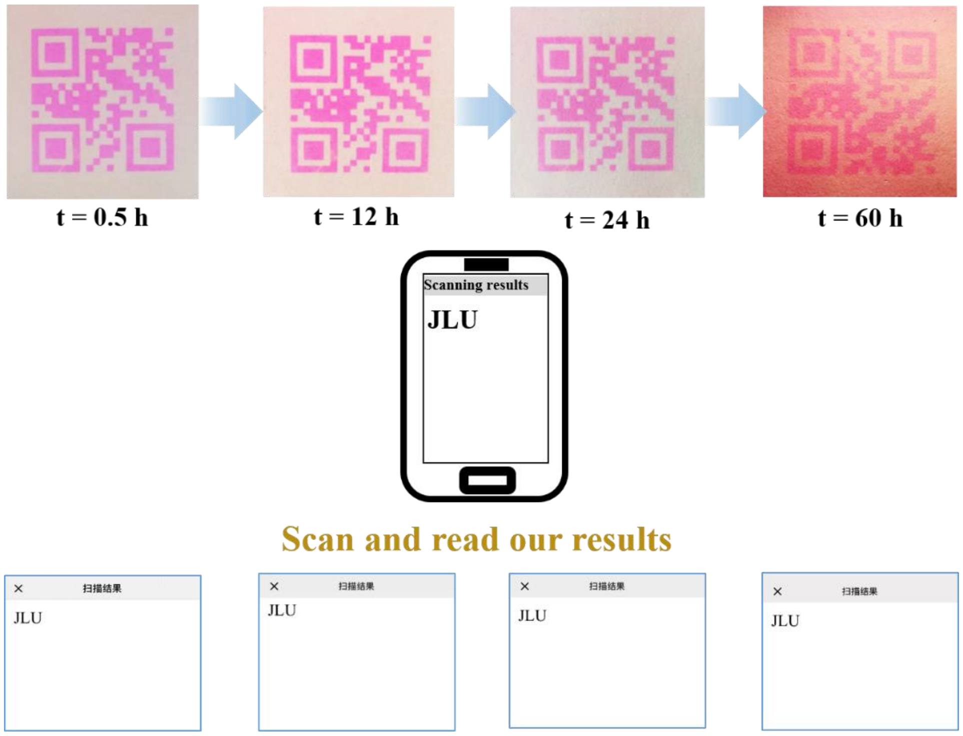

Fig. S16 QR-Code images on **LCM** maintaining in indoor environment (R.H. ≈ 23%) after writing for 0.5 h, 12 h, 24 h and 60 h, and the corresponding read results (Its size was 5 x 5 cm).

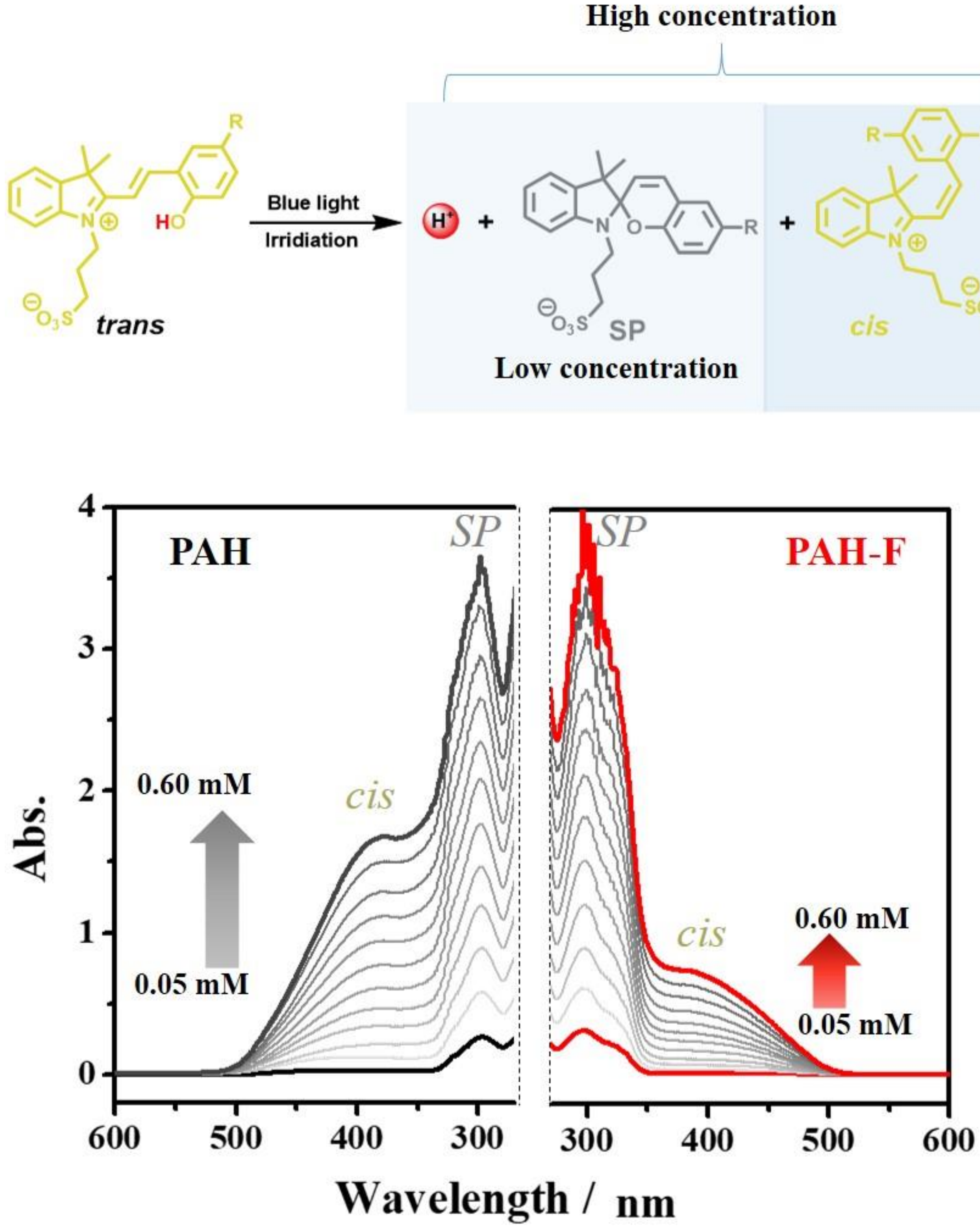

Fig. S17 Structure illustration of photoacid at different concentrations (above) upon irradation with blue light, and UV-vis spectra of PAH and PAH-F in MeOH solution at different concentrations after irradiation (below).

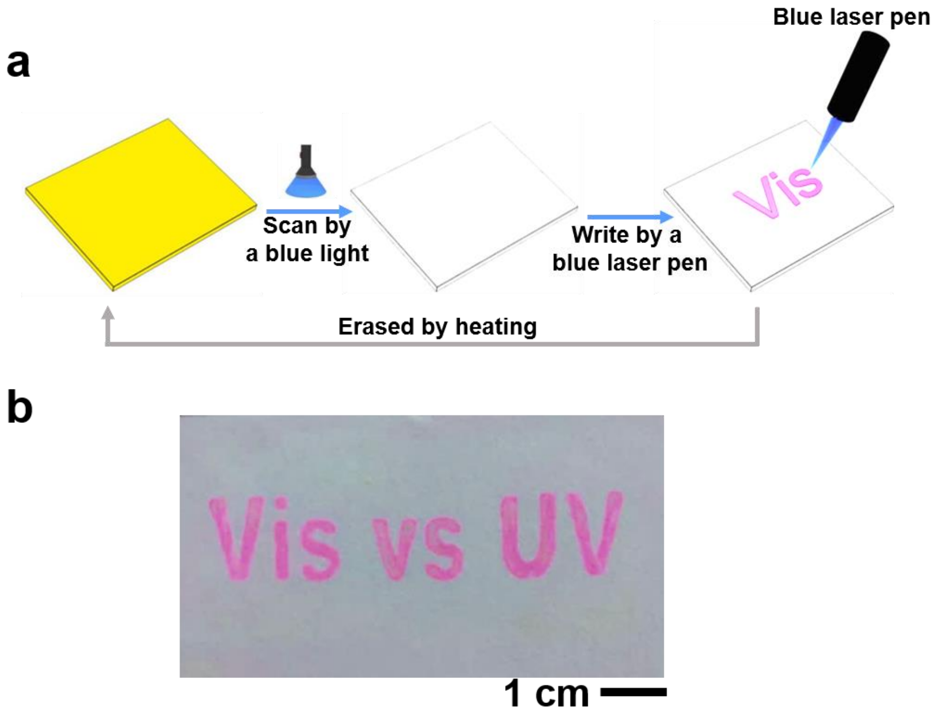

Fig. S18 (**a**) Schematic illustrations of the printed process by a blue laser pen on as-prepared **LCM**. (**b**) An image printed on the **LCM** by a blue laser pen (1.6 W).

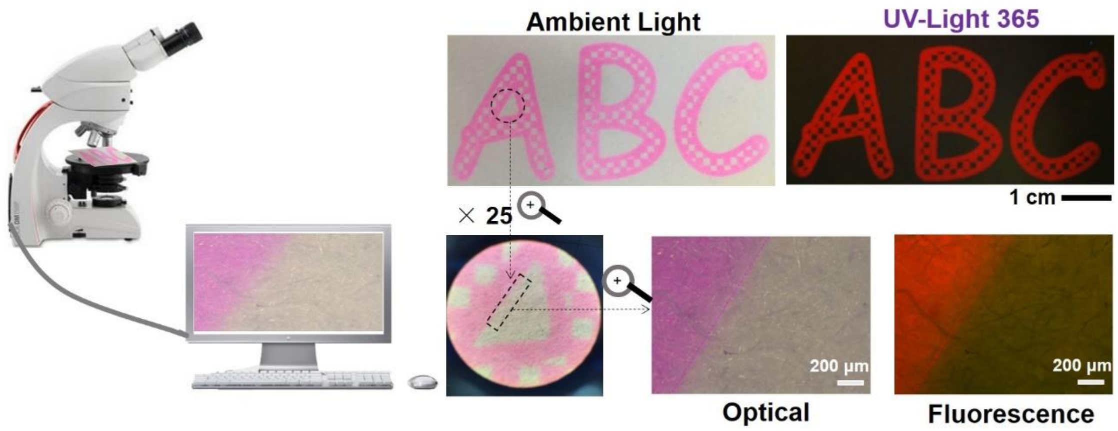

Fig. S19 Left: schematic illustrations of the **LCM** amplification via optical microscopy. Right: images and fluorescent images of letters printed on **LCM** and optical microscopy images of part of the letter, respectively.

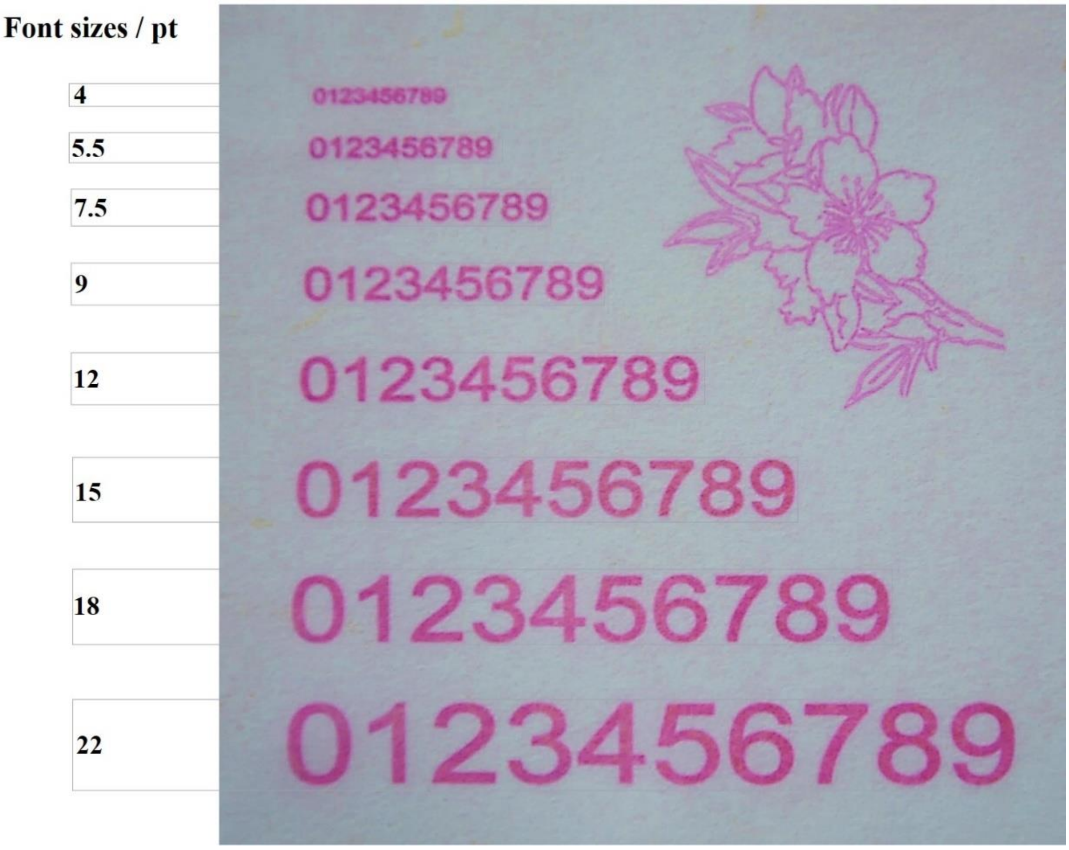

Fig. S20 Numbers with different font sizes printed on **LCM** based on PAH-F & **1**.

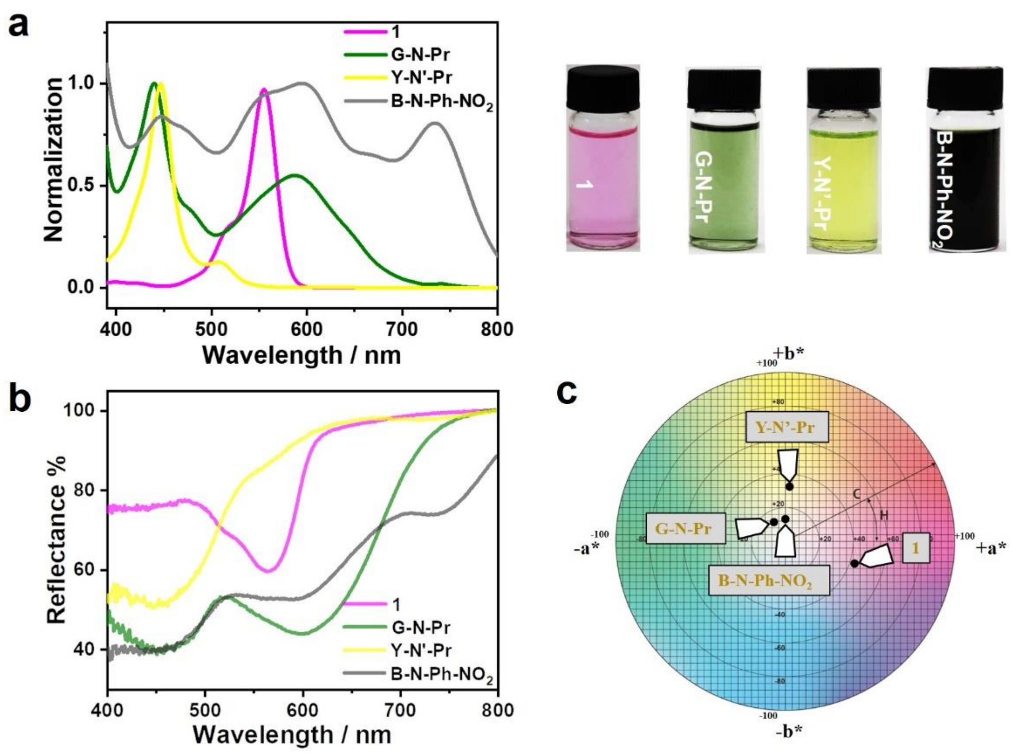

Fig. S21 (**a**) Normalized UV-vis spectra and photographs of four kinds of "inert" acidochromic dyes in solution after treating with acid. (**b**) UV-vis reflection spectra of four kinds of **LCM**s after colouration. (**c**) Plots of corresponding positions of four kinds of **LCM**s after colouration in the CIE 1931 colour space.

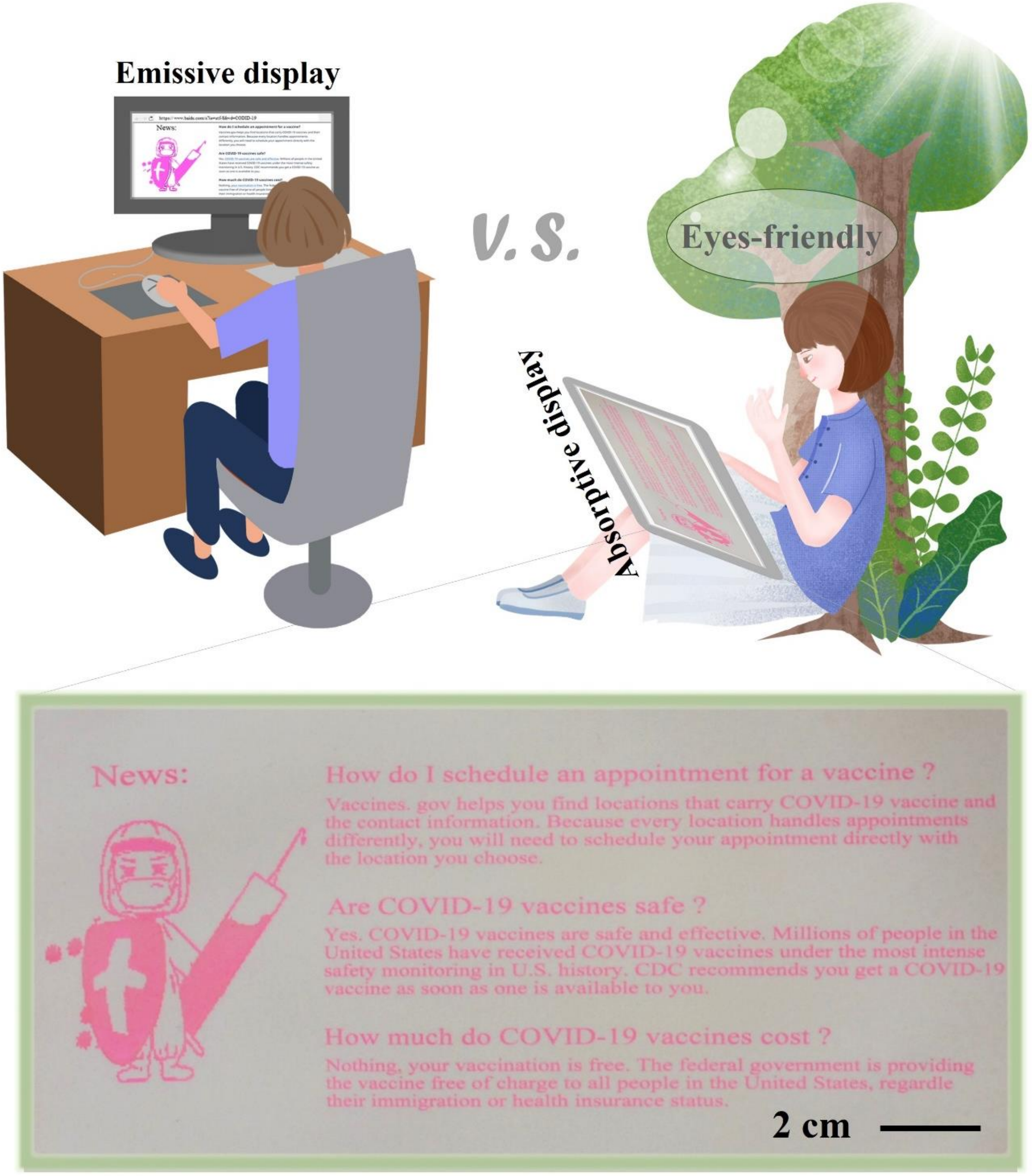

Fig. S22 The prototype of **LCM** in printable/erasable light-absorbing reader.

Supplementary Notes

Note S1 Protonation kinetics of 1—4.

The protonation of **1**—**4** can be described below (Eqn. (1)) according to the reported work[S2]:

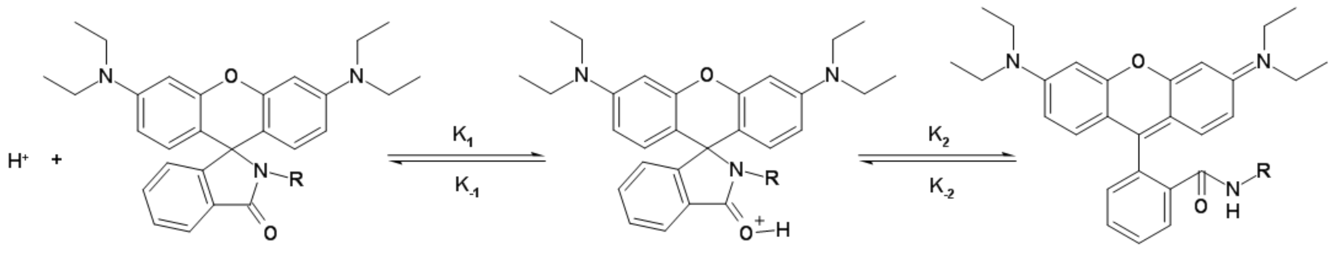

Rh-NR Rh-OH-R Rh-NH-R

$$\text{Rh-NR} + \text{H}^{+} \underset{k_{-1}}{\overset{k_1}{\rightleftharpoons}} \text{Rh-OH-R} \underset{k_{-2}}{\overset{k_2}{\rightleftharpoons}} \text{Rh-NHR} \qquad (1)$$

At the early stage of the reaction, the rate controlling step is the second step — ring-opening step, due to the high concentration (10 equivalent to Rh-B derivatives) of hydrogen ions in the solution. And we assume that $k_2 >> k_{-2}$, thus the rate of the protonation process can be described as below (Eqn. (2)):

$$\frac{\mathrm{d}\ C_t}{\mathrm{dt}} = k_2 C_{Rh-OH-R} \qquad (2)$$

If we assume the first reaction step is in rapid equilibrium, then:

$$\mathrm{k}_1 C_{Rh-NR} C_{H^+} = k_{-1} C_{Rh-OH-R} \qquad (3)$$

Then the rate of the protonation process is:

$$\frac{\mathrm{d}\ C_t}{\mathrm{dt}} = k_2 k_1 C_{H^+} C_{Rh-NR} \ / \ k_{-1} \qquad (4)$$

Defines the constant k to give Eqn. (6):

$$\mathrm{k} = k_2 k_1 C_{H^+} \ / \ k_{-1} \qquad (5)$$

$$\frac{\mathrm{d}\ C_t}{\mathrm{dt}} = k C_{Rh-NR} = k(C_0 - C_t) \qquad (6)$$

Integral the above eqn. (6) to obtain:

$$\ln\left[\left(C_0 - C_t\right)/C_0\right] = kt \qquad (7)$$

Therefore, the data collected at the early stage can be fitted well into a first order reaction where C_0 and C_t are the initial concentration of closed form of **1**—**4** and the concentration at time t of the protonated forms (**Fig. S3**).

Note S2 Ea(s) of 1—4.

To test the temperature-dependent protonation rates of **1**—**4** induced by PAH-F for further evaluating their apparent activation energy (Ea) respectively during this process, continuous light irradiation as well as heating are needed simultaneously while monitoring the UV-vis spectra. As the used light for irradiation would influence the spectral measurement, sampling gradually over time and testing rather than in situ kinetics was adopted. Under this operation condition, the protonation rates of **2**—**4** with fast protonation rates, especially at higher temperatures, cannot be acquired.

Considering the limitation of the experimental operating conditions and the protonation rates of **1** induced by CH_3SO_3H and PAH-F respectively are very similar (**Fig. 1e and S4, Table S2**) as well as similar acidity of PAH-F with CH_3SO_3H, CH_3SO_3H was used for replacing PAH-F to evaluate the Ea(s) for the protonation process of **1**—**4**. In this case, even though the obtained Ea(s) may be not exactly the same by using CH_3SO_3H and PAH-F, their values should be in the same order.

Based on the obtained rate constants in **Table S2**, the energy barrier can be calculated from Arrhenius equation:

$$\ln k = -\frac{Ea}{RT} + \ln \mathrm{A}$$

Thus, the energy barrier is obtained by plotted in the form lnk versus the reciprocal of temperature with the slope of the curves are –Ea / R (**Fig. S6**).

Note S3 Reversibility of 1 & PAH-F under dark or sole heating condition.

Because of the relaxation process of SP-F form to PAH-F taking back the free proton in the solution in the dark, continuous light irradiation is needed to ensure **1** to be maximized pronated. Otherwise, SP-F and **1** will compete for proton in dark conditions, which will lower the pronation of **1**. For a deep understanding, the mixture of PAH-F and **1** was first irradiated till the fully conversion of PAH-F to SP-F (set as time = 0), which could be visualized as the mixture turned to be transparent from yellow. Then the mixture was put in dark and meanwhile the absorption at 554 nm was measured with time (**Fig. S10 solid lines**). As a comparison, the absorption at 554 nm of the transparent mixture kept on irradiating was measured at set intervals (**Fig. S10 dot lines**). For the former situation, large amount of proton released during the irradiation, so for a period of time after the light is off, there is enough proton for **1** to be protonated. As shown in **Fig. S10**, the absorption at 554 nm increased first and the rates increased with temperature. With time going on, the absorption at 554 nm decreased to almost 0. It is inferred that SP-F took the proton from protonated **1** and recovered to PAH-F, which further indicating that the proton transfer is reversible as it will turn to original in dark. While for the latter situation, photoacid existed in the SP-F form all the time, so **1** kept protonated until it reached equilibrium and the time to equilibrium is positively correlated with temperature. Besides, it could be calculated that up to 20% of **1** could be protonated without continuous light irradiation.

Supplementary Tables

Table S1 Summary of crystal data and intensity collection parameters of PAH-F.

Molecule	PAH-F
**Formula**	$C_{21}H_{22}FNO_4S$
**Formula weight**	403.45
**Crystal system, space group**	Triclinic, P-1
**a / Å**	11.3171(4)
**b / Å**	11.3214(4)
**c / Å**	15.1462(6)
**α / º**	89.750(2)
**β / º**	86.463(2)
**γ / º**	87.450(2)
**Volume / Å^3**	1935.00(12)
**Z, Calculated density**	4, 1.385 Mg/m^3
**Absorption coefficient**	1.811 mm^-1
**F_{000}**	848
**Crystal size**	0.200 x 0.180 x 0.150 mm
**Theta range for data collection**	2.923 to 68.540 deg.
**Limiting indices**	-13<=h<=13, -13<=k<=13, -17<=l<=18
**Reflections collected / unique**	43412 / 6992 [R(int) = 0.0964]
**Completeness to theta = 67.679**	98.4 %
**Refinement method**	Full-matrix least-squares on F^2
**Data / restraints / parameters**	6992 / 0 / 511
**Goodness-of-fit on F^2**	1.130
**Final R indices [I>2sigma(I)]**	R1 = 0.0640, wR2 = 0.1387
**R indices (all data)**	R1 = 0.0913, wR2 = 0.1564
**Extinction coefficient**	n/a
**Largest diff. peak and hole**	228.d -0.538 e.A^-3
**CCDC No.**	1950835

Table S2 Rate constants of **1** at different temperatures switched by PAH-F and CH_3SO_3H.

	**25 °C**	**35 °C**	**45 °C**	**55 °C**
$\mathbf{k_{(PAH-F)}}$ / s^{-1}	5.17×10^{-5}	1.68×10^{-4}	4.97×10^{-4}	0.831×10^{-3}
$\mathbf{k_{(CH_3SO_3H)}}$ / s^{-1}	$(7.44\pm0.08)\times10^{-5}$	$(1.97\pm0.09)\times10^{-4}$	$(5.36\pm0.19)\times10^{-4}$	$(1.37\pm0.17)\times10^{-3}$

Table S3 A summary of the calculated Gibbs free energies of the protonation of **1**—**4**.

**Rh-NR**	$\mathbf{\Delta G_i}$	$\mathbf{\Delta G_{ii}}$ (kcal / mol)	$\mathbf{\Delta G_{iii}}$
**1**	-156.85	62.74	-82.87
**2**	-157.10	22.64	-43.29
**3**	-157.52	22.42	-44.23
**4**	-152.33	17.45	-61.99

Table S4 The average value of thickness of filter paper, PEG passivated filter paper and **LCM** (chose 3 groups of samples and each sample was tested 10 times). The below SEM pictures just took one group for examples.

Content	Average thickness / um		
	Filter paper	Filter paper / PEG	**LCM**
Sample 1	92	113	124
Sample 2	89	108	103
Sample 3	86	113	104
Average value	89	111	110

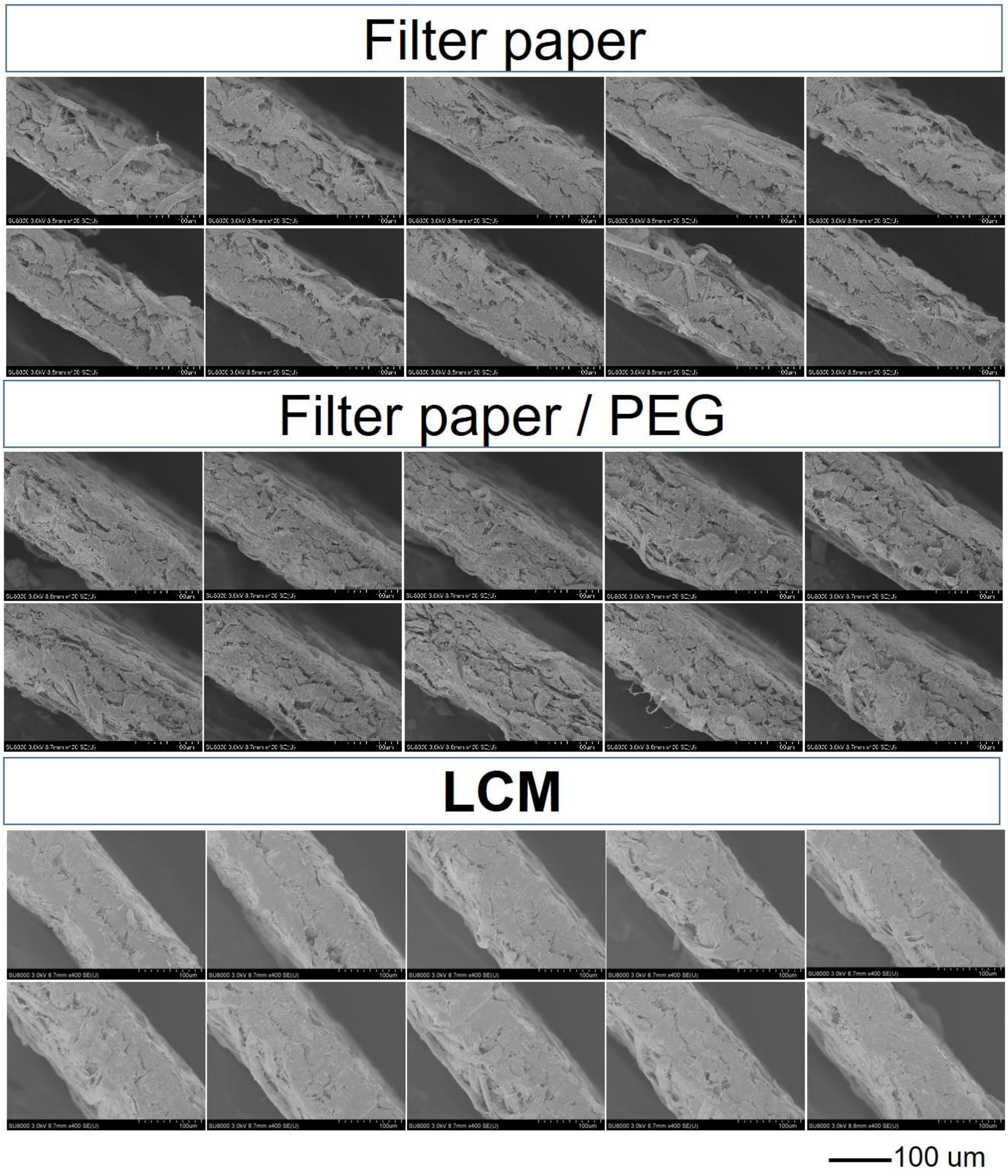

Table S5 Comparion of different reported visible-light-response color-switching materials with those described here.

Photochromic materials	Respond time	Optical memory	Reversibility	Colour change	Reference
Extended azobenzene	20 min	–	4	Orange – Yellow	Ref S8
	–		4	Yellow – Red	Ref S9
	30 s		100[a]	Yellow – Red	Ref S10
Extended schiff base	–	20 min	8	Yellow – Red	Ref S11
		6 min	8		Ref S12
Extended diarylethene (DAE)	–	–	–	Yellow – Green	Ref S13
	–				Ref S14
	1 min				Ref S15
DAE & $NaYF_4$	–	–	–	Yellow – Red	Ref S16
DAE & Triplet sensitizer	20 min	–	8[a]	Yellow – Red	Ref S17
	5 min		3		Ref S18
	12 min		–		Ref S19
Donor–Acceptor Stenhouse Adduct	30 min	–	0	Red – Colourless	Ref S20
Thieno[3,2-b]phosphole oxides	–	–	10	Yellow – Orange	Ref S21
Donor–Acceptor Dihydropyrenes	~ 3 min	–	–	Gray – Green / Blue – Cyan	Ref S22
Coumarin-Diene	–	–	100	Orange – Yellow	Ref S23
Nucleobase	30 s	–	30[a]	Yellow – Red	Ref S24
Binaphthyl-Bridged Imidazole Dimer	> 15 min	–	< 20[a]	Gray – Yellow	Ref S25
MOF	60 s	12 h	~ 4	Yellow – Green	Ref S26
SnO_{2-x} / MB	–	4 h	–	Blue – Colourless	Ref S27
Ag-TiO_2 nanoparticles	60 min	–	–	Yellow – Dark blue	Ref S28
Phosphomolybdate	90 min	–	~ 6	Yellow – Blue	Ref S29
Photoacid / pH-sensitive dyes	2 s	5 h[b]	> 100	Yellow – Coloured	Ref S7
**Photoacid / pH-sensitive dyes**	**Instantly**	**> 60 h**	**> 20**	**White – Coloured**	**This work**

[a] measurement in solution; [b] the material protected with a yellow covering film

Calculated Structures by DFT

The structure for immediate 1-OH

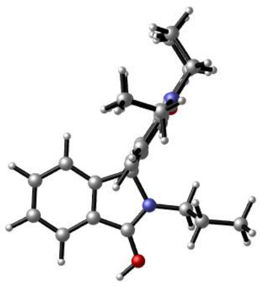

O	-0.10066100	-1.70869600	0.31413100
O	0.00209200	4.37188800	1.66053000
N	4.63338000	-2.14885800	0.02037200
N	-4.85826400	-1.94456500	0.36001900
N	-0.00301200	2.16169500	1.13783500
C	3.51097100	-1.35204000	-0.03846100
C	2.21515500	-1.88161000	0.14252800
H	2.04180700	-2.93766300	0.29516100
C	1.09270200	-1.05659500	0.12388800
C	-1.27566000	-1.00683700	0.20520200
C	-2.42835100	-1.78309100	0.30363300
H	-2.29031100	-2.84595400	0.44466100
C	-3.70967500	-1.19738200	0.21649100
C	-3.75540400	0.20863700	-0.01273300
H	-4.70167600	0.72115400	-0.11701800
C	-2.59399300	0.95088600	-0.11077200
H	-2.68086500	2.01972100	-0.28303000
C	-1.31547100	0.37636000	-0.00115500
C	-0.05177600	1.20528400	-0.07332300
C	1.17719500	0.32352300	-0.08634000
C	2.46790600	0.84250500	-0.29021300
H	2.58688200	1.90711500	-0.46970700
C	3.60086200	0.05109000	-0.27308700
H	4.55830300	0.52192500	-0.44828600
C	5.97015800	-1.59331300	-0.20983900
H	6.68447000	-2.26953800	0.26740300
H	6.05837600	-0.64028000	0.32226500
C	6.34834600	-1.40977800	-1.68539200
H	7.34649200	-0.96608300	-1.76232400
H	5.64353400	-0.75071500	-2.20046500
H	6.36185800	-2.36853700	-2.21116400
C	4.51932500	-3.59532200	0.22636000
H	3.78100300	-3.78488300	1.01324600
H	5.47747500	-3.94034600	0.62392300
C	4.16755000	-4.40241000	-1.03050000
H	4.06382000	-5.46223200	-0.77547600
H	4.94942800	-4.31164800	-1.78968800
H	3.22520500	-4.06631700	-1.47201300
C	-6.18338000	-1.33333600	0.21829000
H	-6.19542800	-0.37708600	0.75171100
H	-6.89110800	-1.97870200	0.74542000
C	-6.65249400	-1.13602200	-1.22885100
H	-7.63663300	-0.65599800	-1.23975800
H	-6.73615100	-2.09389900	-1.74967300
H	-5.96048000	-0.50326100	-1.79190300
C	-0.06838000	2.23198400	-1.19933800
C	-0.10389800	2.01911800	-2.57075700
H	-0.12370900	1.01462700	-2.97956200
C	-0.11454300	3.13698500	-3.41082300
H	-0.14279100	2.99301800	-4.48603800
C	-0.08927600	4.44158400	-2.89494600
H	-0.09782000	5.28769800	-3.57314200
C	-0.05262100	4.66036100	-1.51969100
H	-0.03219000	5.67001200	-1.12239600
C	-0.04290100	3.53602000	-0.69024400
C	-0.01040200	3.41522300	0.75939200
C	-4.79266500	-3.39447400	0.56334600
H	-4.00705300	-3.61544700	1.29416600
H	-5.73273500	-3.69620300	1.03284000
C	-4.57219300	-4.21927100	-0.71168800
H	-4.50720400	-5.28293400	-0.45979100
H	-3.64655100	-3.93485800	-1.22007900
H	-5.39951400	-4.08763200	-1.41475800
C	0.00070200	1.70210300	2.53277800
H	-0.73499600	2.29847300	3.08047600
H	-0.35779100	0.67041100	2.51604400
C	1.37819200	1.79420900	3.20400500
H	1.71696800	2.83559900	3.18848500
H	2.09991600	1.21173400	2.62339900
C	1.32248700	1.28501000	4.64714800
H	1.01263500	0.23534800	4.68784900
H	2.30614300	1.36141200	5.11884900
H	0.61675100	1.86752000	5.24906600
H	-0.02305900	5.25458100	1.25698600

The structure for immediate 2-OH

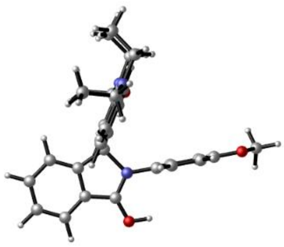

O	-0.04612500	1.66188000	0.43455200	H	6.74304200	2.18321500	0.36878600
O	0.03524600	-4.45493100	-0.64983900	C	6.33228200	2.19911300	-1.75159000
N	-4.79832800	2.00362400	0.52180200	H	7.31763400	1.81752900	-2.03895000
N	4.68330700	2.18204500	0.16753400	H	6.35231900	3.28899300	-1.83956400
N	-0.00481400	-2.15411500	-0.26220500	H	5.59827300	1.81462600	-2.46554600
C	-3.66377800	1.35235500	0.09079700	C	-0.09305900	-1.43605600	-2.49898100
C	-2.37175500	1.80662900	0.43433100	C	-0.14816400	-0.79197700	-3.72835800
H	-2.21352600	2.70755500	1.01045100	H	-0.17424700	0.29047000	-3.79210700
C	-1.23621400	1.10882600	0.03112900	C	-0.16843400	-1.57904600	-4.88460400
C	1.13076400	1.15125400	-0.05461400	H	-0.21146300	-1.09404900	-5.85456800
C	2.26620900	1.89250700	0.26218300	C	-0.13429200	-2.98069500	-4.82088400
H	2.11689200	2.78994500	0.84605900	H	-0.15119200	-3.55988100	-5.73765700
C	3.54588100	1.48307200	-0.17203000	C	-0.07920300	-3.63235800	-3.59114600
C	3.60345000	0.30389800	-0.97096200	H	-0.05250700	-4.71408700	-3.52238500
H	4.54632000	-0.05237400	-1.36217400	C	-0.05981300	-2.83466200	-2.44523200
C	2.45732900	-0.40702500	-1.27362900	C	-0.00574200	-3.20811600	-1.04672900
H	2.55161100	-1.30010600	-1.88430900	C	4.60208300	3.41818900	0.95066700
C	1.18325700	-0.01507100	-0.82612800	H	3.89098400	3.27391000	1.77126700
C	-0.05771300	-0.82210900	-1.10627000	H	5.57778400	3.56558300	1.42126200
C	-1.30232000	-0.05606200	-0.74171200	C	4.22882700	4.66957600	0.14494800
C	-2.59020100	-0.48830700	-1.10481600	H	4.15665600	5.53468200	0.81230000
H	-2.69613300	-1.37960600	-1.71607100	H	3.26597000	4.54947600	-0.35959700
C	-3.73625100	0.17958300	-0.71618000	H	4.98437700	4.88891000	-0.61448800
H	-4.69146700	-0.20626200	-1.04416000	C	0.04092200	-2.20732500	1.16873300
C	-6.13276500	1.54670100	0.12124100	C	-1.15388900	-2.20481000	1.90645900
H	-6.83855900	1.93458100	0.86044800	C	1.27458200	-2.22384600	1.82610500
H	-6.17843600	0.45575200	0.20675200	C	-1.10477200	-2.23147000	3.29116700
C	-6.57279500	1.98346400	-1.28205100	H	-2.10719500	-2.18087400	1.39062100
H	-7.56520600	1.57718200	-1.50367300	C	1.32597200	-2.25148100	3.21877300
H	-5.88095700	1.62476400	-2.04954100	H	2.19308400	-2.21267700	1.24974000
H	-6.62793300	3.07305700	-1.35777300	C	0.13353700	-2.25381300	3.95821000
C	-4.70567500	3.22151700	1.33159700	H	-2.01494100	-2.23477100	3.88060400
H	-3.93159200	3.08288900	2.09438200	H	2.28989400	-2.26899000	3.71071500
H	-5.64900800	3.31900400	1.87553300	H	0.07074400	-4.51471700	0.32420700
C	-4.43887200	4.50688300	0.53737500	O	0.06899300	-2.27843000	5.31079400
H	-4.35139900	5.35681800	1.22216400	C	1.28966200	-2.29557700	6.05571900
H	-5.25509000	4.71570500	-0.15977400	H	1.88805200	-1.40016100	5.85696600
H	-3.51132100	4.43818600	-0.03790100	H	0.99564400	-2.31035600	7.10478800
C	6.00225000	1.76459400	-0.31768500	H	1.87999000	-3.18994400	5.82971800
H	6.08901800	0.67684800	-0.22549300				

The structure for immediate 3-OH

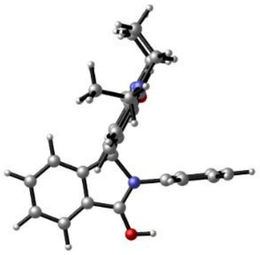

O	0.00005000	-1.68889600	0.19901400
O	-0.00027200	4.41371900	1.31474200
N	4.74363400	-2.06668800	-0.04584500
N	-4.74351000	-2.06678300	-0.04612700
N	-0.00009600	2.12039300	0.86736100
C	3.60675800	-1.30071400	-0.17735500
C	2.32032000	-1.83817000	0.04634800
H	2.16664900	-2.88155400	0.28359900
C	1.18431500	-1.03836500	-0.04345200
C	-1.18420400	-1.03839500	-0.04356300
C	-2.32020700	-1.83822200	0.04618300
H	-2.16652100	-2.88159100	0.28349300
C	-3.60663700	-1.30080200	-0.17761700
C	-3.67124600	0.07758800	-0.53598300
H	-4.62056800	0.54820400	-0.75132100
C	-2.52463300	0.84468100	-0.62129900
H	-2.62442500	1.89141500	-0.89231400
C	-1.24331300	0.32061900	-0.37364600
C	0.00004000	1.16964800	-0.39857900
C	1.24341300	0.32066400	-0.37349500
C	2.52474300	0.84477200	-0.62099100
H	2.62453900	1.89152200	-0.89194400
C	3.67136700	0.07769600	-0.53563000
H	4.62069500	0.54834900	-0.75086300
C	6.06773900	-1.51746100	-0.35458300
H	6.80071800	-2.14359900	0.16067400
H	6.15701400	-0.52141500	0.09234100
C	6.40947400	-1.45643600	-1.84883800
H	7.39922400	-1.00865600	-1.98649900
H	5.68455300	-0.85205600	-2.40142900
H	6.42487400	-2.45616100	-2.29187300
C	4.66014400	-3.48290400	0.32208700
H	3.91204100	-3.59982100	1.11332000
H	5.61873900	-3.75537400	0.77201000
C	4.35238300	-4.43714800	-0.83939600
H	4.27746200	-5.46430600	-0.46742100
H	5.14327000	-4.40719500	-1.59394100
H	3.40738000	-4.18402300	-1.32839700
C	-6.06762100	-1.51756500	-0.35485100
H	-6.15687400	-0.52149600	0.09202200
H	-6.80058900	-2.14366900	0.16046400
C	-6.40940000	-1.45661800	-1.84909900
H	-7.39915600	-1.00885100	-1.98675700
H	-6.42480400	-2.45636500	-2.29208200
H	-5.68449700	-0.85226100	-2.40174000
C	0.00010900	2.23932500	-1.48190200
C	0.00022100	2.07321600	-2.86090400
H	0.00028000	1.08367400	-3.30486400
C	0.00028000	3.21946200	-3.66286600
H	0.00038000	3.11014000	-4.74261300
C	0.00021000	4.50732600	-3.10494600
H	0.00026000	5.37421500	-3.75662500
C	0.00008700	4.68018200	-1.72298300
H	0.00003700	5.66697500	-1.27383900
C	0.00004400	3.52772700	-0.93465700
C	-0.00011400	3.38335900	0.50681400
C	-4.66000900	-3.48298200	0.32186900
H	-3.91193900	-3.59985300	1.11314100
H	-5.61862100	-3.75545100	0.77175600
C	-4.35217600	-4.43726200	-0.83956500
H	-4.27724700	-5.46440500	-0.46755000
H	-3.40715700	-4.18413400	-1.32853200
H	-5.14302900	-4.40735400	-1.59414700
C	-0.00020400	1.65704400	2.22491100
C	-1.21993000	1.42034300	2.87050000
C	1.21940300	1.41998600	2.87057300
C	-1.21188100	0.94910900	4.18301300
H	-2.15178500	1.60136100	2.34665900
C	1.21112800	0.94875800	4.18309500
H	2.15134900	1.60076100	2.34681500
C	-0.00042800	0.71314400	4.83706900
H	-2.15245000	0.76618500	4.69191600
H	2.15161700	0.76557000	4.69204900
H	-0.00052100	0.34587900	5.85835700
H	-0.00037900	4.13868700	2.25077900

The structure for immediate 4-OH

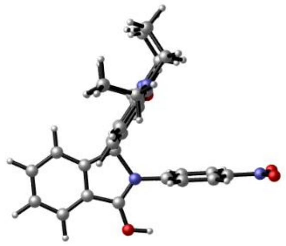

O	0.04059500	-0.16178800	-1.69870000
O	-0.04253100	0.52055400	4.48038500
N	-4.69062700	-0.52322200	-2.12571400
N	4.79185700	-0.23849900	-2.05257500
N	-0.00659900	0.24849600	2.15344600
C	-3.55454300	-0.67610600	-1.36428500
C	-2.27301800	-0.37412500	-1.87547200
H	-2.12188000	-0.05779600	-2.89802100
C	-1.13911100	-0.48619600	-1.07645300
C	1.22808400	-0.41191200	-1.05811500
C	2.36561600	-0.23182800	-1.83962100
H	2.21151500	0.07088200	-2.86583700
C	3.65508200	-0.45228800	-1.30720400
C	3.72226300	-0.89811500	0.04602200
H	4.67518500	-1.11122300	0.51008500
C	2.57469800	-1.07383200	0.79475800
H	2.67615500	-1.41094700	1.82191200
C	1.28881600	-0.83437300	0.27581300
C	0.04343000	-0.96281100	1.10560400
C	-1.19413900	-0.91604200	0.25532700
C	-2.46993500	-1.24319200	0.75075300
H	-2.56572500	-1.59468400	1.77360700
C	-3.61417500	-1.13772800	-0.01625500
H	-4.55838000	-1.41717700	0.43005400
C	-6.00872800	-0.90639900	-1.60849800
H	-6.75213900	-0.36313600	-2.19759200
H	-6.11229800	-0.53886400	-0.58211900
C	-6.31282700	-2.40889500	-1.66340000
H	-7.30379700	-2.60312600	-1.24003200
H	-5.58146200	-2.98812800	-1.09256300
H	-6.30617200	-2.77518900	-2.69364900
C	-4.61098200	-0.05779300	-3.51363200
H	-3.88684700	0.76169900	-3.57136700
H	-5.58129200	0.38073700	-3.76172300
C	-4.26273100	-1.14288300	-4.54085600
H	-4.19175200	-0.70040200	-5.53990800
H	-5.03102800	-1.92050600	-4.56877200
H	-3.30538700	-1.61999100	-4.31267000
C	6.12086600	-0.55262400	-1.51666700
H	6.19744600	-0.16563400	-0.49491800
H	6.84421200	0.01547000	-2.10713500
C	6.49453000	-2.04019700	-1.54536900
H	7.48793700	-2.18213600	-1.10720500
H	6.51781200	-2.42209600	-2.56973800
H	5.78366300	-2.64414700	-0.97431300
C	0.07427100	-2.10716300	2.10743900
C	0.12526500	-3.47102500	1.84968700
H	0.14830200	-3.84708800	0.83271100
C	0.14591200	-4.34741100	2.93981600
H	0.18497000	-5.41666100	2.75851300
C	0.11697200	-3.87744600	4.26191000
H	0.13396900	-4.58533400	5.08333200
C	0.06671700	-2.51112400	4.52646600
H	0.04396500	-2.12931500	5.54092500
C	0.04624300	-1.64750700	3.42906800
C	-0.00525200	-0.20051000	3.38867900
C	4.70723700	0.21730600	-3.44330500
H	3.93238000	0.98735400	-3.51646500
H	5.65121500	0.71789400	-3.67548000
C	4.44894600	-0.89121000	-4.47198200
H	4.36648000	-0.45756100	-5.47399300
H	3.52135900	-1.42980400	-4.25787800
H	5.26723100	-1.61635700	-4.48320000
C	-0.07807300	1.62209200	1.76669300
C	1.10156300	2.31466100	1.46471600
C	-1.33176300	2.23667900	1.65287800
C	1.02769200	3.64248600	1.05555500
H	2.06044400	1.81854900	1.55344400
C	-1.40817600	3.56532800	1.24640300
H	-2.23236200	1.67766000	1.87725300
C	-0.22601700	4.24164300	0.95430600
H	1.91964700	4.20723200	0.81914800
H	-2.36056100	4.06987400	1.15267900
H	-0.06859000	1.47765300	4.29889400
N	-0.30464500	5.64659100	0.52096000
O	-1.41972400	6.16004300	0.43249700
O	0.74884700	6.23143900	0.27013500

The structure for immediate 1-TS

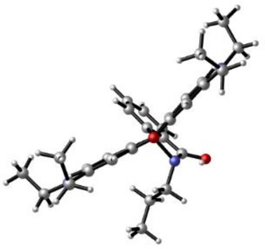

O	-0.16964800	-1.60555200	0.82680700	H	2.78618400	-4.50492700	-0.88072200
O	-1.27912000	3.73313600	1.91863800	C	-6.21357300	-1.31199500	0.04189300
N	4.45458100	-2.42127700	-0.00175500	H	-6.27661800	-0.26581500	0.35982300
N	-4.92157900	-1.86399900	0.46191900	H	-6.98536300	-1.84316500	0.60503500
N	-0.06146900	2.02370000	1.41205200	C	-6.50865400	-1.42524300	-1.45931800
C	3.37226100	-1.57693400	-0.05690900	H	-7.48004600	-0.97187200	-1.68262600
C	2.09470400	-1.97689500	0.40030000	H	-6.54111100	-2.47115100	-1.77662800
H	1.89948000	-2.97847600	0.75655300	H	-5.75030200	-0.91255500	-2.05792500
C	1.02252600	-1.09427900	0.37902200	C	-0.04145600	2.31148700	-0.98464200
C	-1.33280600	-0.95698200	0.47901400	C	0.07016800	2.23417300	-2.36903200
C	-2.49517700	-1.70499400	0.62038700	H	0.26106000	1.28604900	-2.85879700
H	-2.38643000	-2.72009000	0.97594400	C	-0.06966100	3.40684900	-3.11688600
C	-3.75386600	-1.15560000	0.28470000	H	0.03292700	3.35900400	-4.19613100
C	-3.75241900	0.16619700	-0.24373500	C	-0.35372900	4.64411300	-2.51188600
H	-4.67391000	0.63411100	-0.56115300	H	-0.46114100	5.52988200	-3.12774200
C	-2.57739800	0.88792800	-0.36577100	C	-0.50816200	4.73323500	-1.13564400
H	-2.63952800	1.89427300	-0.76673900	H	-0.73753400	5.67333400	-0.64736300
C	-1.32823100	0.36001600	0.00111700	C	-0.32730900	3.56066000	-0.38695700
C	-0.04083500	1.14414100	0.00451300	C	-0.53352900	3.29023000	0.99836800
C	1.13353100	0.22922500	-0.08118800	C	-4.90193900	-3.23055700	0.99123200
C	2.39728800	0.60338300	-0.57817500	H	-4.18497400	-3.28020500	1.81762300
H	2.53221700	1.59826100	-0.98910700	H	-5.88389200	-3.41815400	1.43421600
C	3.47971300	-0.25507900	-0.58088500	C	-4.59083100	-4.32112200	-0.04224200
H	4.41304200	0.09986000	-0.99499500	H	-4.56168100	-5.30033200	0.44697500
C	5.76705300	-2.00648100	-0.50978200	H	-3.62318000	-4.15392600	-0.52393700
H	6.51442500	-2.62479800	-0.00586400	H	-5.35630300	-4.35531400	-0.82244600
H	5.96334500	-0.97768800	-0.19097000	H	-1.05295200	2.51414700	2.26789900
C	5.93951400	-2.13166900	-2.02885700	C	1.20230400	2.03650000	2.25229400
H	6.93375900	-1.77754500	-2.32023600	H	0.88498000	2.20921600	3.28357400
H	5.19640400	-1.53708000	-2.56786800	H	1.59129800	1.01856100	2.19670300
H	5.84149100	-3.17138900	-2.35308200	C	2.26483900	3.06197500	1.85516000
C	4.31929400	-3.79079700	0.50577500	H	2.54969700	2.93430800	0.80768500
H	3.69020400	-3.77666300	1.40208100	H	1.85307100	4.07411100	1.95211500
H	5.31062400	-4.10886600	0.83878000	C	3.50381500	2.92723000	2.74657100
C	3.77077300	-4.80217100	-0.50885200	H	3.25031900	3.05739900	3.80379000
H	3.67291100	-5.78539100	-0.03722100	H	3.96874000	1.94290300	2.62867900
H	4.44087800	-4.90292100	-1.36700300	H	4.24809700	3.68400300	2.48450800

The structure for immediate 2-TS

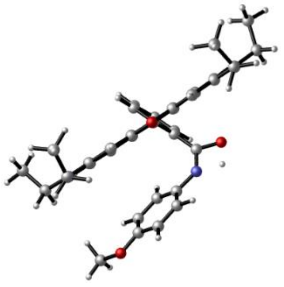

O	-0.87471700	1.94033900	-0.19447200	H	-7.43688400	0.86940300	-1.60343200
O	-2.00535000	-3.69905000	-1.00439900	C	-7.44954100	0.65227700	0.54713400
N	3.69935900	3.06148000	0.41488600	H	-8.34820000	0.02801800	0.56360700
N	-5.52189600	1.30889500	-0.94730500	H	-7.74952900	1.68475200	0.74384200
N	-0.22842900	-2.74146800	-1.58703100	H	-6.79456700	0.32708300	1.36017400
C	2.67618900	2.18095300	0.56119800	C	-0.40401000	-1.93830900	1.60967500
C	1.36127000	2.48758700	0.12534900	C	-0.11652800	-1.99949800	2.98160100
H	1.10800700	3.43949200	-0.31819000	H	0.01960400	-1.07593400	3.53443100
C	0.34874400	1.56238900	0.26590100	C	-0.03432800	-3.22383200	3.64354800
C	-1.95604400	1.12092400	-0.08095200	H	0.18658800	-3.24530400	4.70565100
C	-3.14985000	1.62508700	-0.55237700	C	-0.24979300	-4.41200500	2.94491700
H	-3.13839400	2.62014500	-0.97281100	H	-0.19595400	-5.36725300	3.45640500
C	-4.33792000	0.85473500	-0.46129100	C	-0.54213700	-4.37015900	1.58349500
C	-4.23510400	-0.43451400	0.17025800	H	-0.71481800	-5.28794900	1.03164400
H	-5.11594500	-1.04640100	0.30157500	C	-0.60277500	-3.14554100	0.90499800
C	-3.03894600	-0.91645400	0.62485900	C	-0.90576400	-3.17638500	-0.54585000
H	-3.01228400	-1.89313900	1.09142400	C	-5.62590000	2.63573100	-1.57859500
C	-1.82818100	-0.17133600	0.50907300	H	-4.75637500	2.78467500	-2.22494000
C	-0.56197700	-0.60147100	0.96607900	H	-6.49457000	2.60536800	-2.23933500
C	0.53724800	0.27627600	0.85369100	C	-5.76640600	3.79841800	-0.58963900
C	1.86699400	-0.01822900	1.27716300	H	-5.80169100	4.74295900	-1.14109200
H	2.07414000	-0.98889700	1.70989100	H	-4.92315900	3.84149400	0.10495800
C	2.88604500	0.88436500	1.15118700	H	-6.68605700	3.71265100	-0.00517600
H	3.86860800	0.60035400	1.49997200	H	-1.35728700	-3.29551600	-2.06072100
C	5.05260800	2.76384300	0.91897100	C	1.10977000	-2.33404900	-1.74250700
H	5.73988800	3.41286400	0.37320700	C	1.40609700	-1.37483200	-2.72607200
H	5.31860500	1.73990800	0.64218100	C	2.16448300	-2.89838600	-1.01291500
C	5.22567900	2.97740900	2.42639400	C	2.71536300	-0.97754600	-2.95516500
H	6.25006000	2.72279600	2.71518100	H	0.59510600	-0.94505000	-3.30534200
H	4.54360600	2.34787500	3.00447400	C	3.48396900	-2.49773100	-1.23346700
H	5.04380000	4.01929200	2.70250200	H	1.96125500	-3.66852600	-0.27641000
C	3.48509100	4.38074900	-0.20464300	C	3.76665300	-1.53155600	-2.20688700
H	2.82092600	4.25919100	-1.06451500	H	2.94804700	-0.23590300	-3.71232800
H	4.44777300	4.70253300	-0.60708000	H	4.27600800	-2.95485800	-0.65316800
C	2.93940000	5.44642000	0.75227100	O	5.01606700	-1.07300500	-2.50786900
H	2.78161200	6.38099900	0.20535500	C	6.12128300	-1.59771200	-1.77591700
H	3.64196000	5.64401300	1.56586100	H	7.00626400	-1.09670700	-2.16892500
H	1.98476200	5.14332600	1.19083800	H	6.02974900	-1.38469700	-0.70439300
C	-6.76312100	0.52419700	-0.81673500	H	6.22281900	-2.67930300	-1.92173800
H	-6.54607800	-0.52325800	-1.04201800				

The structure for immediate 3-TS

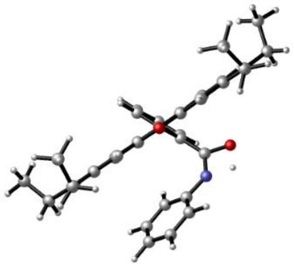

O	0.35854600	-1.91333700	-0.19085600
O	2.14697200	3.54854900	-1.11068400
N	-4.33829200	-2.54667300	0.11813900
N	5.08852800	-1.78692500	-0.59960300
N	0.35006900	2.72522500	-1.81757400
C	-3.24350700	-1.76544800	0.30373000
C	-1.93920500	-2.22056700	-0.02035600
H	-1.75415900	-3.21294200	-0.40496400
C	-0.84981000	-1.39479300	0.15962200
C	1.50711200	-1.20108700	-0.02681900
C	2.67423400	-1.84467800	-0.38054900
H	2.59230300	-2.85279200	-0.75987400
C	3.92524200	-1.19294200	-0.22804800
C	3.90980400	0.12944300	0.33993900
H	4.83727200	0.65465400	0.51724700
C	2.73939100	0.74981300	0.67895600
H	2.77954200	1.74453000	1.10451400
C	1.47005900	0.12455600	0.49787100
C	0.22493900	0.69935200	0.83897300
C	-0.94759500	-0.06984000	0.67939700
C	-2.26713400	0.37454500	0.98887300
H	-2.40433100	1.38011900	1.36592700
C	-3.36138400	-0.42770600	0.82269700
H	-4.33175000	-0.03067800	1.08375800
C	-5.68720400	-2.09087200	0.50229400
H	-6.39570000	-2.69219300	-0.07044700
H	-5.82404000	-1.05971200	0.16580500
C	-5.99354200	-2.21556700	1.99843200
H	-7.00734100	-1.85282300	2.19365700
H	-5.29961600	-1.62579800	2.60370900
H	-5.93477600	-3.25578900	2.32859600
C	-4.21622600	-3.91180000	-0.42211800
H	-3.48828700	-3.90100200	-1.23791600
H	-5.17763800	-4.15996000	-0.87626800
C	-3.84407700	-4.97296200	0.61903400
H	-3.74411200	-5.94568800	0.12783200
H	-4.61501700	-5.05995300	1.38915300
H	-2.89522000	-4.74104600	1.11043400
C	6.38929800	-1.11515700	-0.42692200
H	6.28624200	-0.06502400	-0.71190600
H	7.07123400	-1.55997300	-1.15444400
C	6.98209100	-1.23665900	0.98083300
H	7.93273500	-0.69630600	1.02347500
H	7.17338200	-2.28067700	1.24217600
H	6.31558600	-0.81246900	1.73692000
C	0.15808500	2.07467100	1.41300900
C	-0.23095900	2.23029700	2.75215900
H	-0.50600000	1.35269300	3.32767300
C	-0.23996700	3.48702900	3.35572500
H	-0.54272800	3.58107800	4.39340900
C	0.15160500	4.61333800	2.63173100
H	0.15417900	5.59267200	3.09843400
C	0.54932100	4.47738800	1.30363200
H	0.86093000	5.34567000	0.73308100
C	0.53885100	3.22141000	0.68212200
C	0.96733500	3.15332000	-0.73518300
C	5.10220200	-3.15377400	-1.14883300
H	4.28061800	-3.25212900	-1.86437500
H	6.02285900	-3.25359900	-1.72694400
C	5.03187900	-4.26147400	-0.09185200
H	5.02327700	-5.23706800	-0.58744000
H	4.12793900	-4.18264200	0.51810100
H	5.89703600	-4.22718800	0.57522800
H	1.56461800	3.14874500	-2.20432200
C	-0.99794500	2.43540100	-2.09097400
C	-1.27877400	1.49127400	-3.09074200
C	-2.05831100	3.10532700	-1.45716900
C	-2.59859500	1.20802900	-3.43678500
H	-0.45362200	0.98860500	-3.58525300
C	-3.37562100	2.80977500	-1.80641900
H	-1.84944800	3.86381100	-0.71037000
C	-3.65338400	1.86212100	-2.79440000
H	-2.80308400	0.47458500	-4.21105400
H	-4.18767300	3.33445500	-1.31172200
H	-4.68072500	1.64218000	-3.06740100

The structure for immediate 4-TS

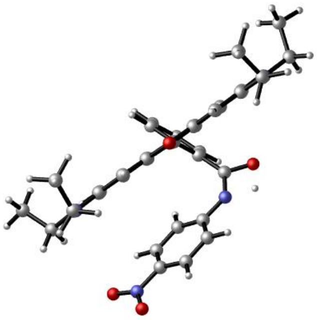

O	-0.85004500	1.92928100	-0.02315000
O	-2.19646100	-3.64786800	-1.15370800
N	3.77309700	2.73006000	0.71358000
N	-5.48592600	1.57607200	-0.99309600
N	-0.42190600	-2.64533400	-1.63172700
C	2.68653300	1.92659000	0.83497600
C	1.40383800	2.33115700	0.38159900
H	1.22730200	3.30445700	-0.05246000
C	0.33232500	1.46792600	0.46792100
C	-1.97692300	1.16608800	0.01346900
C	-3.12171100	1.75142000	-0.48398500
H	-3.04204300	2.76081500	-0.86026400
C	-4.34985000	1.04039700	-0.47824600
C	-4.34057700	-0.27886200	0.09826800
H	-5.25664100	-0.84760600	0.16627000
C	-3.19168000	-0.84121800	0.58098600
H	-3.23631300	-1.83465000	1.00987200
C	-1.94116800	-0.15537300	0.54983200
C	-0.72029300	-0.66906200	1.04097400
C	0.42460800	0.15589000	1.02188200
C	1.71578800	-0.22315500	1.49461700
H	1.84653400	-1.20699400	1.92709000
C	2.79292300	0.61416500	1.41717900
H	3.74064500	0.26682800	1.80239400
C	5.11343500	2.28412900	1.13795400
H	5.83447100	2.86657800	0.56091500
H	5.25210300	1.24362000	0.83173700
C	5.39214500	2.45478400	2.63473100
H	6.39868700	2.09028600	2.86157400
H	4.68077200	1.89212900	3.24561200
H	5.33718100	3.50596800	2.92964200
C	3.65318900	4.09487500	0.17109700
H	3.00747700	4.06857900	-0.71157000
H	4.64389400	4.38262700	-0.18515600
C	3.14376900	5.12840800	1.18136900
H	3.06318700	6.10486800	0.69416600
H	3.83003300	5.22538300	2.02659800
H	2.15793300	4.86072000	1.57106900
C	-6.76848500	0.84958400	-0.95864000
H	-6.59027600	-0.19727800	-1.21723700
H	-7.38276600	1.25933600	-1.76286100
C	-7.51882200	0.95727700	0.37291700
H	-8.44769600	0.38159000	0.31558400
H	-7.77604700	1.99520400	0.59925200
H	-6.92583100	0.56419900	1.20339000
C	-0.65368300	-2.04694600	1.61072200
C	-0.43180900	-2.19955100	2.98703100
H	-0.28352200	-1.31571700	3.59834300
C	-0.42784800	-3.46197300	3.57938600
H	-0.25480400	-3.55212300	4.64667000
C	-0.66013400	-4.59900200	2.80561100
H	-0.66683500	-5.58280600	3.26243700
C	-0.89370400	-4.46838400	1.43880200
H	-1.08156200	-5.34490400	0.82834500
C	-0.87304100	-3.20523200	0.83077100
C	-1.12300900	-3.15390100	-0.62773000
C	-5.49630900	2.93612200	-1.55979700
H	-4.59307700	3.07137400	-2.16125800
H	-6.33568900	2.98291700	-2.25643500
C	-5.62214300	4.05379700	-0.51855600
H	-5.58902800	5.02498400	-1.02165900
H	-4.80750900	4.02035000	0.20991800
H	-6.56841600	3.98483700	0.02401500
H	-1.53287700	-3.17019600	-2.16553300
C	0.90078600	-2.23097900	-1.75286100
C	1.20975800	-1.33904400	-2.79880800
C	1.93439800	-2.70653100	-0.92040200
C	2.50909700	-0.89443000	-2.98460600
H	0.41257200	-0.99407000	-3.44761100
C	3.23413300	-2.25514500	-1.09474600
H	1.71825400	-3.43267900	-0.14604700
C	3.50888800	-1.34583400	-2.11850700
H	2.75563900	-0.19727100	-3.77484000
H	4.03544900	-2.60260500	-0.45559900
N	4.86709900	-0.84898800	-2.28003800
O	5.70924300	-1.16084600	-1.42998100
O	5.11672700	-0.13173400	-3.25424500

Supplementary References

S1 M. J. Frisch, Trucks, G. E. H. B. Schlegel, G. E. Scuseria, M. A. Robb, J. R. Cheeseman, et al. Gaussian 09, Revision D.01. Gaussian, Inc. Wallingford CT, **2013**.

S2 W. L. Czaplyski, G. E. Purnell, C. A. Roberts, R. M. Allred & E. J. Harbron, *Org. Biomol. Chem.* **2014**, *12*, 526–533.

S3 B. H. S. T. Da Silva, B. A. Bregadiolli, C. F. O. Graeff & L. C. Da Silva-Filho, *ChemPlusChem.* **2017**, *82*, 261–269.

S4 Z. Shi, P. Peng, D. Strohecker & Y. Liao, *J. Am. Chem. Soc.* **2011**, *133*, 14699−14703.

S5 Y. Suzuki & H. Takahashi, *Chem. Pharm. Bull.* **1983**, *31*, 1751−1753.

S6 L. Sheng, M. Li, S. Zhu, H. Li, G. Xi, Y. G. Li, Y. Wang, Q. Li, S. Liang, K. Zhong & S. X.-A. Zhang, *Nat. Commun.* **2014**, *5*, 3044.

S7 T. Zhang, L. Sheng, J. Liu, L. Ju, J. Li, Z. Du, W. Zhang, M. Li & S. X.-A. Zhang, *Adv. Funct. Mater.* **2018**, *28*, 1705532.

S8 P. Weis, D. Wang & S. Wu, *Macromolecules* **2016**, *49*, 6368-6373.

S9 S. Li, G. Han & W. Zhang, *Macromolecules* **2018**, *51*, 4290−4297.

S10 P. Lentes, E. Stadler, F. Röhricht, A. Brahms, J. Gröbner, F. D. Sönnichsen, G. Gescheidt & R. Herges, *J. Am. Chem. Soc.* **2019**, *141*, 13592–13600.

S11 H. Sun, S.-S. Sun, F.-F. Han, Z.-H. Ni, R. Zhang & M.-D. Li, *J. Mater. Chem. C* **2019**, *7*, 7053-7060.

S12 H. Sun, J.-Y. Li, F.-F. Han, R. Zhang, Y. Zhao, B.-X. Miao & Z.-H. Ni, *Dyes Pigments* **2019**, 167, 143-150.

S13 H. Xi, Z. Zhang, W. Zhang, M. Li, C. Lian, Q. Luo, H. Tian & W. H. Zhu, *J. Am. Chem. Soc.* **2019**, *141*, 18467-18474.

S14 G. Liu, Y. M. Zhang, C. Wang & Y. Liu, *Chem. Eur. J.* **2017**, *23*, 14425-14429.

S15 Z. Li, Y.-J. Hu, K. Zhang, Y. Zhang, Q.-Q. Hu, X.-J. Zhang, X.-K. Zhang & Y.-P. Zhu, *Dyes Pigments* **2020**, *182*, 108686.

S16 C. J. Carling, J. C. Boyer & N. R. Branda, *J. Am. Chem. Soc.* **2009**, *131*, 10838–10839.

S17 S. Fredrich, R. Göstl, M. Herder, L. Grubert & S. Hecht, *Angew. Chem. Int. Ed.* **2016**, *55*, 1208–1212.

S18 Z. Zhang, W. Wang, P. Jin, J. Xue, L. Sun, J. Huang, J. Zhang & H. Tian, *Nat. Commun.* **2019**, *10*, 4232.

S19 Z. Zhang, J. Zhang, B. Wu, X. Li, Y. Chen, J. Huang, L. Zhu & H. Tian, *Adv. Optical Mater.* **2018**, *6*, 1700847.

S20 G. Sinawang, B. Wu, J. Wang, S. Li & Y. He, *Macromol. Chem. Phys.* **2016**, *217*, 2409–2414.

S21 N. M. W. Wu, M. Ng, W. H. Lam, H. L. Wong & V. W. W. Yam, *J. Am. Chem. Soc.* **2017**, *139*, 15142-15150.

S22 K. Klaue, W. Han, P. Liesfeld, F. Berger, Y. Garmshausen & S. Hecht, *J. Am. Chem. Soc.* **2020**, *142*, 11857-11864.

S23 M. T. Richers, D. Du Tran, J. Wachtveitl & G. C. Ellis-Davies, *Chem. Commun.* **2018**, *54*, 4983-4986.

S24 S. Ogasawara, S. Ito, H. Miyasaka & M. Maeda, *Chem. Lett.* **2010**, *39*, 956−957.

S25 A. Kometani, Y. Inagaki, K. Mutoh & J. Abe, *J. Am. Chem. Soc.* **2020**, *142*, 7995–8005.

S26 B. Garai, A. Mallick & R. Banerjee, *Chem. Sci.* **2016**, *7*, 2195-2200.

S27 D. Han, B. Jiang, J. Feng, Y. Yin & W. Wang, *Angew. Chem. Int. Ed.* **2017**, *56*, 7792-7796.

S28 D. M. Tobaldi, M. J. Hortigüela Gallo, G. Otero-Irurueta, M. K. Singh, R. C. Pullar, M. P. Seabra & J. A. Labrincha, *Langmuir* **2017**, *33*, 4890-4902.

S29 Z. Wang, R. Zhang, Y. Ma, L. Zheng, A. Peng, H. Fu & J. Yao, *J. Mater. Chem.* **2010**, *20*, 1107-1111.
